# Starch biosynthesis in guard cells has features of both autotrophic and heterotrophic tissues

**DOI:** 10.1093/plphys/kiac087

**Published:** 2022-03-03

**Authors:** Sabrina Flütsch, Daniel Horrer, Diana Santelia

**Affiliations:** 1 Institute of Integrative Biology, ETH Zürich, 8092 Zürich, Switzerland; 2 Department of Plant and Microbial Biology, University of Zürich, 8008 Zürich, Switzerland

## Abstract

The pathway of starch synthesis in guard cells (GCs), despite the crucial role starch plays in stomatal movements, is not well understood. Here, we characterized starch dynamics in GCs of Arabidopsis (*Arabidopsis thaliana*) mutants lacking enzymes of the phosphoglucose isomerase-phosphoglucose mutase-ADP-glucose pyrophosphorylase starch synthesis pathway in leaf mesophyll chloroplasts or sugar transporters at the plastid membrane, such as glucose-6-phosphate/phosphate translocators, which are active in heterotrophic tissues. We demonstrate that GCs have metabolic features of both photoautotrophic and heterotrophic cells. GCs make starch using different carbon precursors depending on the time of day, which can originate both from GC photosynthesis and/or sugars imported from the leaf mesophyll. Furthermore, we unravel the major enzymes involved in GC starch synthesis and demonstrate that they act in a temporal manner according to the fluctuations of stomatal aperture, which is unique for GCs. Our work substantially enhances our knowledge on GC starch metabolism and uncovers targets for manipulating GC starch dynamics to improve stomatal behavior, directly affecting plant productivity.

## Teaser

Guard cells synthesize starch using carbon precursors originating in the plastid and/or imported from the cytosol depending on the time of the day.

## Introduction

Starch is the most abundant form in which plants store carbohydrates. It is composed of homopolymers of glucose (Glc), which form dense, insoluble, semi-crystalline granules within plastids. The way starch is synthesized and metabolized may vary upon the tissue in which it is found. In autotrophic tissues, such as the leaf mesophyll, starch is gradually formed during the day using a portion of the carbon fixed through photosynthesis; at night, starch is degraded to support non-photosynthetic leaf metabolism and the export of sucrose (Suc) (Smith and Zeeman, 2020). In heterotrophic tissues, such as seeds, tubers, or roots, starch accumulates often for many months and is synthesized using precursors derived from Suc imported from source tissues (MacNeill et al., 2017).

In the leaf mesophyll, starch is the end-product of a biosynthetic pathway that takes place exclusively within the chloroplast and is directly linked to the Calvin–Benson–Bassham (CBB) cycle by means of the phosphoglucose isomerase (PGI) enzyme. PGI generates Glc-6-phosphate (G6P) from the primary photosynthetic product fructose-6-phosphate (F6P). Phosphoglucose mutase (PGM) further converts G6P into Glc-1-phosphate (G1P), which is ultimately used for the ATP-consuming generation of the activated glucosyl donor ADPGlc by the ADPGlc pyrophosphorylase (AGPase; Pfister and Zeeman, 2016). Each enzymatic step of this linear oligosaccharide synthesis is essential, as the loss of either PGI (Yu et al., 2000), PGM (Caspar et al., 1985), or AGPase (Lin et al., 1988) results in leaf chloroplasts nearly devoid of starch and stunted plant growth. The subsequent biosynthetic steps required for starch synthesis involve several starch synthases, starch branching, and starch debranching enzymes (Pfister and Zeeman, 2016).

Although several biochemical steps of starch synthesis occurring in photosynthetic leaves are conserved in heterotrophic tissues, some are specific to sink organs. For instance, in the endosperm, starch is formed following the incorporation of Suc-derived sugar metabolites entering the plastid via a G6P/phosphate translocator (GPT). This transmembrane protein was initially detected in the plastidial envelope membranes of maize (*Zea mays*) endosperm (Kammerer et al., 1998). Subsequently, GPT cDNAs were isolated from different plant species and in planta functional studies confirmed GPT function as G6P transporter, including Arabidopsis (*Arabidopsis thaliana*) (Niewiadomski et al., 2005) and grapevine (*Vitis vinifera*) (Noronha et al., 2015). Besides G6P, some plant species can also import cytosolic G1P into plastids, as shown for potato (*Solanum tuberosum*) tubers (Fettke et al., 2010). Alternatively, in heterotrophic tissues of rice (*Oryza sativa*), wheat (*Triticum aestivum*), and potato, G1P is directly added to elongating glucan chains via the α-glucan phosphorylase (PHS1) (Satoh et al., 2008; Tickle et al., 2009; Fettke et al., 2010). Lastly, it has been shown in cereal endosperm that ADPGlc is produced in the cytosol and subsequently imported into the amyloplast via Brittle1 (BT1) (Kirchberger et al., 2007).

Starch is also present in guard cells (GCs) (Lloyd, 1908) that surround the stomatal pore on the leaf epidermis of vascular plants. Through reversible changes in turgor pressure, GCs regulate stomatal aperture facilitating CO_2_ uptake for photosynthesis, while limiting water loss through transpiration. These highly specialized cells possess several characteristics of heterotrophic tissues, such as high respiratory rates (Willmer and Fricker, 1996), fewer chloroplasts (Willmer and Fricker, 1996), low levels of CBB cycle enzyme ribulose-1,5-biphosphate carboxylase/oxygenase (RubisCO) (Outlaw, 1989; Reckmann et al., 1990), calling into question the ability of GCs to perform photosynthesis. Even though the electron transport chain in GC chloroplasts is functional and RubisCO is a major sink for the end product of electron transport (Lawson et al., 2003), it was recently reported that GC photosynthesis is limited and mitochondria are the major source of ATP (Lim et al., 2022). Unlike mesophyll cell (MC) chloroplasts, GC chloroplasts import cytosolic ATP through nucleotide transporter proteins to compensate for their limited photosynthesis (Lim et al., 2022).

In GCs, starch shows a distinct temporal pattern of accumulation and degradation that differs in several aspects from that of MCs. In Arabidopsis, GC starch is abundant during the night and is rapidly mobilized within 1-h of illumination, after which starch progressively accumulates until the middle of the night (Horrer et al., 2016). Starch breakdown in GCs coincides with stomatal opening (Horrer et al., 2016) and yields Glc to maintain sugar homeostasis needed for fast changes in GC turgor pressure (Flütsch et al., 2020a). Interestingly, in isolated GCs, where there is no connection with the mesophyll, starch accumulation is limited compared with intact GCs (Flütsch et al., 2020a). Furthermore, GCs rely on the uptake of mesophyll-derived Glc as a main carbon source for starch formation (Flütsch et al., 2020b; Lim et al., 2022). These findings suggest that majority of carbon precursors for GC starch synthesis derives from mesophyll photosynthesis rather than GC autonomous photosynthesis. However, the relative contribution of each pathway to the pool of accumulated GC starch and the enzymatic steps involved remain unknown.

In this study, we investigated the early steps of starch biosynthesis in Arabidopsis GCs. We report the functional characterization of genes related to the classical pathway of starch biosynthesis, such as *PGI*, *PGM*, and the *AGPase*, as well as genes linked to uptake of sugars to the chloroplast, including the *GPTs*. Although GCs are limited in photosynthetic activities (Outlaw, 1989; Reckmann et al., 1990; Lim et al., 2022), here we reveal that GCs synthesize starch using carbon substrates derived both from GC and MC photosynthesis. Our data substantially advance the knowledge of starch synthesis in GCs and provide a genetic framework for future manipulations of GC starch metabolism to improve stomatal function and plant productivity.

## Results

### *pgi* mutants are unable to synthesize starch in GCs at the beginning of the day

**Figure 1:**
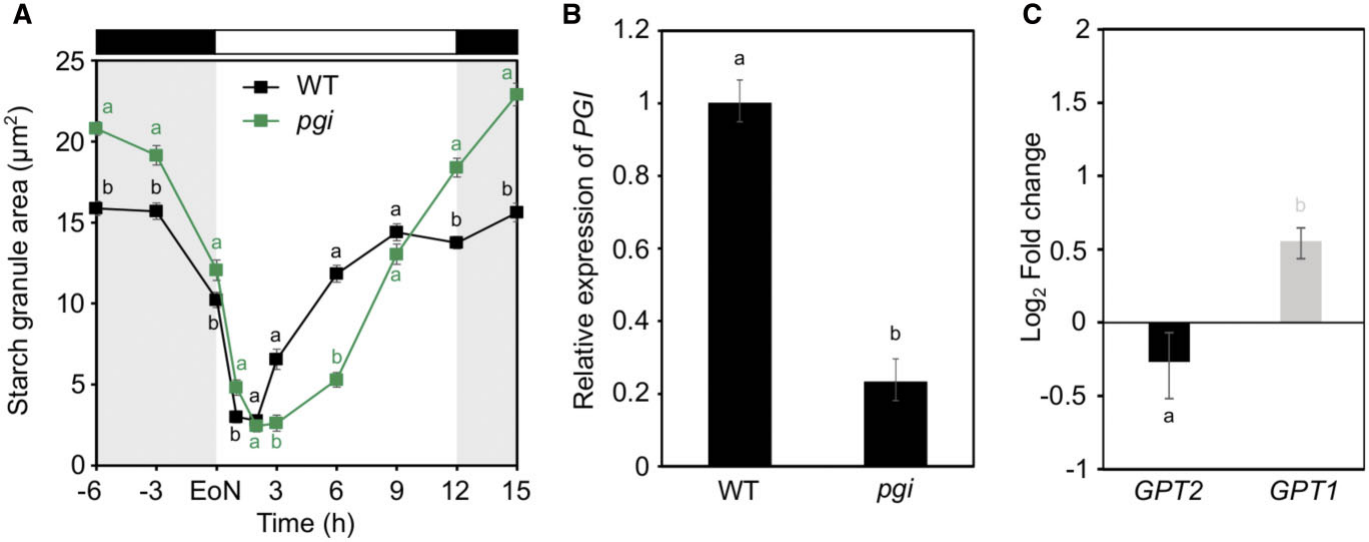
GC starch contents and *GPT* gene expression in *pgi* mutants. A, Starch dynamics in GCs of intact leaves of WT and *pgi* plants over the 24 h diel cycle. Plants were illuminated with 150 µmol m^−2^ s^−1^ of white light. Data from three independent experiments are shown; means ± sem; *n* = 120 individual GCs per genotype and time point. Letters indicate significant statistical difference between genotypes for the given time point for *P* < 0.05 determined by one-way ANOVA with post hoc Tukey’s test. B, *PGI* gene expression in intact rosette leaves of WT and *pgi* plants at the EoN. C, *GPT1* and *GPT2* gene expression in *pgi* GC-enriched epidermal peels relative to WT GC-enriched epidermal peels at the EoN. B and C, Data from two independent experiments are shown; means ± fold change range; *n* = 6. *ACT2* was used as a housekeeping gene for normalization. For details about fold change and error calculations refer to “Materials and methods.” Primer sequences and efficiencies are given in Supplemental Table S7. Letters indicate significant statistical difference between genes for *P* < 0.05 determined by one-way ANOVA with post hoc Tukey’s test. A–C, WT.

In the leaf mesophyll, the first committed step of starch synthesis is the PGI-mediated conversion of the CBB cycle intermediate F6P to G6P (Stitt and Zeeman, 2012). A previous study reported that GCs of Arabidopsis *pgi* mutants have similar amounts of starch to that of wild-type (WT), suggesting that PGI is not required for GC starch synthesis. However, starch granules were only visualized at the end of the day (EoD) (Azoulay-Shemer et al., 2016). Here, we examined stomatal starch levels in EMS-mutagenized *pgi-1* plants (herein named *pgi*; Yu et al., 2000) throughout the 24 h day/night cycle (Figure 1A). GCs of *pgi* mutants contained elevated amounts of starch during the night and had significantly more starch at the end of the night (EoN) compared with WT (Figure 1A). Upon light exposure, starch was rapidly degraded and almost fully consumed within the first hour of light in both *pgi* and WT GCs, with comparable negative slope-derived starch synthesis rates (WT_0__–__1_, −0.61; *pgi*_0-1_, *−*0.56; Supplemental Table S1). However, while WT GCs substantially accumulated starch starting at 2 h of light (WT_2__–__3_, 1.27; Supplemental Table S2), starch synthesis was negligible in *pgi* GCs between 2 and 3 h and remained low until 6 h into the day (Figure 1A;*pgi*_2__–__3_, 0.13; Supplemental Table S1). Following this lag phase, starch synthesis rates and starch accumulation substantially increased in *pgi* mutant GCs (Figure 1A and Supplemental Table S1) to reach markedly higher starch amounts by the EoD compared with WT (Figure 1A).

Although the *pgi* mutant had reduced expression of *PGI*, it was not a complete knockout (Figure 1B), possibly explaining why *pgi1* GCs still accumulated some starch. This finding is consistent with previous reports indicating ∼5% remaining PGI enzyme activity (Yu et al., 2000), and ∼25% of WT starch levels in the same mutant line (Niewiadomski et al., 2005). Another explanation is that the PGI reaction may be circumvented by import of cytosolic G6P via GPT transporters at the inner chloroplast membrane. The Arabidopsis genome encodes two *GPT* genes, *GPT1* and *GPT2*. Constitutive expression of *GPTs* in *pgi* leaves was indeed shown to rescue the starch deficient phenotype of *pgi* mutant (Niewiadomski et al., 2005). Furthermore, an early study demonstrated the presence of a G6P transport activity in isolated GC chloroplasts from pea (*Pisum sativum*) (Overlach et al., 1993). We detected ∼1.5-fold transcriptional upregulation of *GPT1* gene in GC-enriched epidermal peels of *pgi* mutants relative to WT (Figure 1C), pointing toward a role for GPT1 in *pgi* GC starch accumulation. By contrast, *GPT2* was expressed in *pgi* GCs to similar or even reduced levels than in WT (Figure 1C).

### Loss of GPT1 perturbs GC starch accumulation during the second half of the day

**Figure 2:**
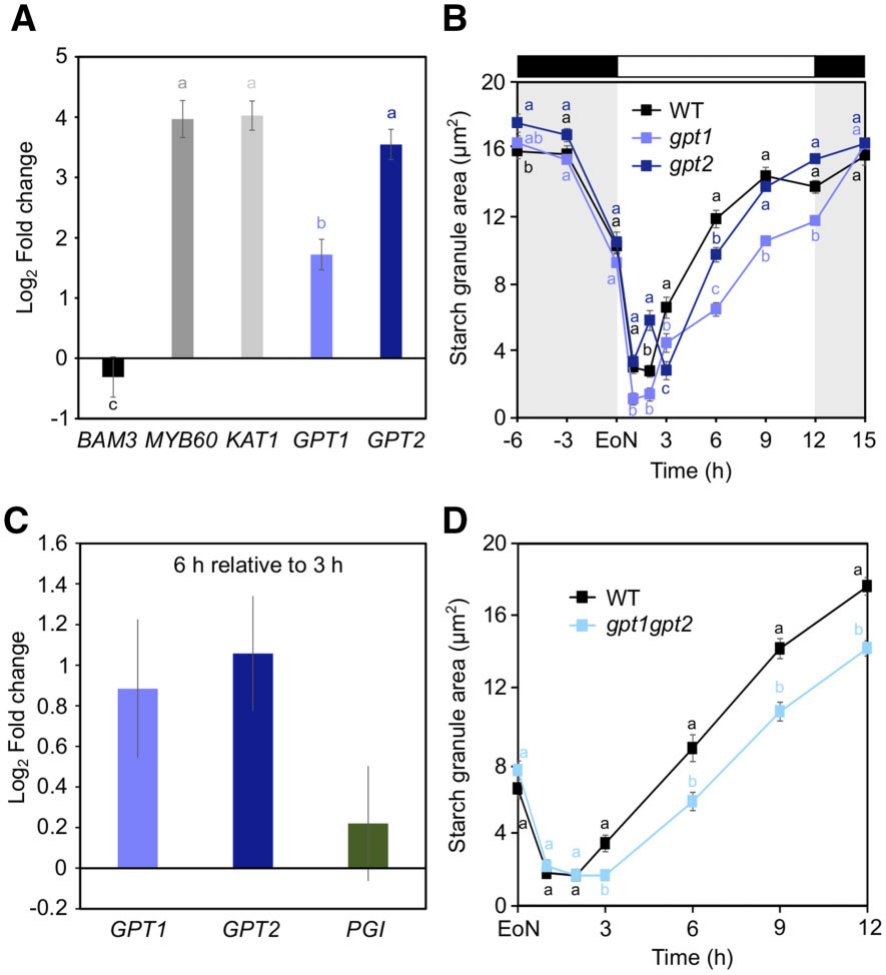
GPT gene expression in GCs and GC starch contents in *gpt* mutants. A, *GPT1* and *GPT2* gene expression in WT GC-enriched epidermal peels relative to WT intact rosette leaves at the EoN. *KAT1* and *MYB60* were used as markers for GC-specific expression, while *BAM3* was used as a leaf-specific marker. B, Starch dynamics in GCs of intact leaves of WT, *gpt1* and *gpt2* plants over the 24 h diel cycle. WT data are the same as in Figure 1A. C, *GPT1*, *GPT2*, and *PGI* gene expression in WT GC-enriched epidermal peels at 6 h relative to 3 h into the day. D, Starch dynamics in GCs of intact leaves of WT and *gpt1gpt2* plants over the 12 h light period. A and C, Data from two independent experiments are shown; means ± fold change range; *n* = 6. *ACT2* was used as a housekeeping gene for normalization. For details about fold change and error calculations, refer to “Materials and methods.” Primer sequences and efficiencies are given in Supplemental Table S7. Letters indicate significant statistical difference between genes for *P* < 0.05 determined by one-way ANOVA with post hoc Tukey’s test. B and D, Plants were illuminated with 150 µmol m^−2^ s^−1^ of white light. Data from three independent experiments are shown; means ± sem; *n* = 120 individual GCs per genotype and time point. Letters indicate significant statistical difference between genotypes for the given time point for *P* < 0.05 determined by one-way ANOVA with post hoc Tukey’s test.

The phenotype of *pgi* mutants prompted us to examine whether GPTs were involved in GC starch metabolism. The *GPT1* gene was approximately four-fold upregulated in WT GC-enriched epidermal peels relative to leaves (Figure 2A), in line with an earlier report indicating 10-fold upregulation of *GPT1* in GC protoplasts compared with MC protoplasts (Niewiadomski et al., 2005). *GPT2* was expressed to similar levels as the GC marker genes *K^+^ inward rectifier channel 1* (*KAT1*) and *Myb transcription factor 60* (*MYB60*), therefore displaying a more pronounced preferential GC expression than *GPT1* (Figure 2A).

We obtained homozygous T-DNA mutant lines of *GPT1* (*gpt1-3;* herein named *gpt1*; SALK_021762) and *GPT2* (*gpt2*; GABIKAT_454H06) and quantified stomatal starch amounts during the 24 h light/dark cycle (Figure 2B). Although WT and *gpt1* plants had similar overall pattern of GC starch synthesis and degradation, *gpt1* GCs contained significantly less starch throughout the day (Figure 2B). Starch was broken down to a similar extent in both genotypes during the first hour of light (Figure 2B; WT_0__–__1_, −0.61 and *gpt1*_0__–__1_, −0.82; Supplemental Table S1), followed by comparable starch accumulation rate between 2 and 3 h of light (Figure 2B; WT_2__–__3_, 1.27; *gpt1*_2__–__3_, 1.00; Supplemental Table S1). After 3 h into the day, however, *gpt1* GCs accumulated less starch compared with WT, and reached the EoD with significantly reduced starch amounts (Figure 2B and Supplemental Table S1). These data suggest that import of cytosolic G6P to chloroplasts *via* GPT1 during the second half of the day contributes to starch accumulation in GCs. This idea is further supported by the approximately two-fold upregulation of *GPT1* gene at 6 h compared with 3 h of light in WT GC-enriched epidermal peels (Figure 2C). Interestingly, during the first 3 h of darkness, *gpt1* GCs accumulated starch at very high rates, reaching WT levels, after which starch amounts remained similar to that of WT for the remainder of the night (Figure 2B and Supplemental Table S1).

Although *GPT2* was highly expressed in GCs relative to leaves (Figure 2A), stomatal starch contents in *gpt2* mutants were WT-like throughout the majority of the 24 h light/dark cycle (Figure 2B). *gpt2* GCs displayed an unusual pattern of net increase and decrease in GC starch contents between 1 and 3 h of light (Figure 2B and Supplemental Table S1). However, after 6 h, GC starch amounts in *gpt2* were only mildly reduced compared with WT and rose to WT levels after 9 h of light (Figure 2B and Supplemental Table S1). By the EoD and for the entire duration of the night, *gpt2* GCs had mildly elevated starch levels compared with WT (Figure 2B and Supplemental Table S1).

Surprisingly, we also observed induced *GPT2* gene expression at 6 h compared with 3 h (Figure 2C), which did not match GC starch contents in *gpt2* plants. Given that *GPT1* was also highly expressed at this time of the day (Figure 2C), while *PGI* was not (Figure 2C), we suggest that GPT1 activity might compensate for the lack of GPT2. Altogether, these data suggest that GPT1 is the predominant GPT isoform in GCs required to deliver cytosolic G6P to the chloroplast, which is used for GC starch accumulation during the second half of the day.

### *gpt1gpt2* double mutants phenocopy *gpt1* single mutants

The observed formation of starch in *gpt1* GCs could be due to GC photosynthesis and subsequent conversion of F6P into G6P via PGI or import of cytosolic G6P through the closely related GPT2 translocator. To assess the contribution of GPT2 to starch accumulation in *gpt1* mutants, we generated the double mutant *gpt1gpt2* and examined GC starch amounts during the 12 h light phase (Figure 2D). To our surprise, the additional loss of GPT2 in *gpt1* single mutant had no further impact on GC starch accumulation during the day (Figure 2D and for comparison Figure 2B). Starch contents at the EoN were comparable between all genotypes and starch degradation occurred at similar rates until 2 h of light (Figure 2D; WT_0__–__1_, −0.58; WT_1__–__2_, −0.24; *gpt1gpt2*_0__–__1_, −0.64; *gpt1gpt2*_1__–__2_, −0.05; Supplemental Table S2). While WT GCs progressively accumulated starch from 2 h onward, *gpt1gpt2* double mutants showed a short lag phase of starch synthesis between 2 and 3 h (Figure 2D and Supplemental Table S2), after which starch levels started to raise but remained overall lower compared with WT (Figure 2D and Supplemental Table S2). Based on these results, we conclude that it is unlikely GPT1 and GPT2 work redundantly in G6P uptake to GC chloroplasts. GPTs may have distinct roles in GC starch metabolism at different time of the day.

### *gpt1pgi* double mutants are not devoid of GC starch

**Figure 3:**
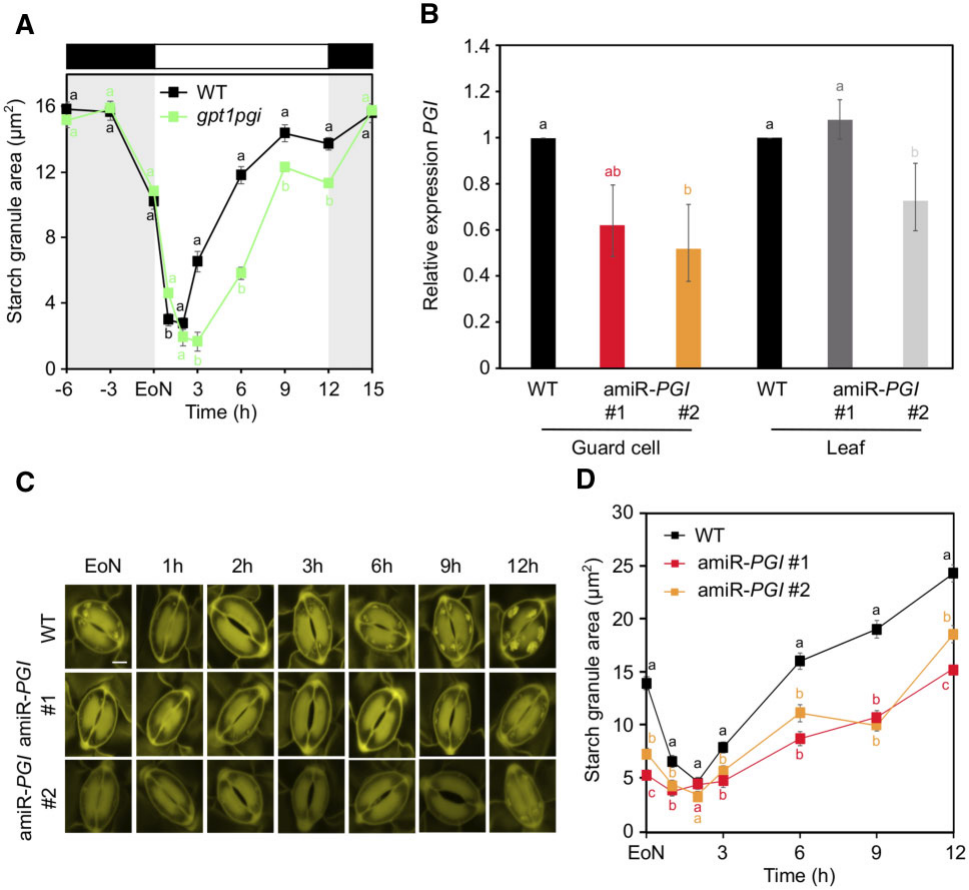
GC starch contents in *gpt1pgi* double mutants and *PGI* silencing lines in *gpt1gpt2* backgrounds. A, Starch dynamics in GCs of intact leaves of WT and *gpt1pgi* plants over the 24-h diel cycle. WT data are the same as in Figure 1A. Data from three independent experiments are shown; means ± sem; *n* = 120 individual GCs per genotype and time point. B, *PGI* gene expression in GC-enriched epidermal peels of artificial microRNA-induced silencing lines of *PGI* in the *gpt1gpt2* mutant background (amiRNA-*PGI*) relative to WT GC-enriched epidermal peels and in intact rosette leaves of amiRNA-*PGI* lines relative to WT intact rosette leaves at EoN. Data from one experiment are shown; means ± fold change range; *n* ≤ 5. Letters indicate significant statistical difference between genes for *P* < 0.05 determined by one-way ANOVA with post hoc Tukey’s test. *ACT2* was used as a housekeeping gene for normalization. For details about fold change and error calculations, refer to “Materials and methods.” Primer sequences and efficiencies are given in Supplemental Table S7. C, Representative confocal images of propidium iodide-stained starch granules in GCs of intact leaves of WT and amiRNA-*PGI* plants over the 12-h light period. Scale bar = 10 µm. D, Starch dynamics in GCs of intact leaves of WT and amiRNA-*PGI* plants over the 12 h light period. Data from one experiment are shown; means ± sem; *n* = 120 individual GCs per genotype and time point. A and D, Plants were illuminated with 150 µmol m^−2^ s^−1^ of white light. Letters indicate significant statistical difference between genotypes for the given time point for *P* < 0.05 determined by one-way ANOVA with post hoc Tukey’s test. A–D, WT.

Given that GPT2 did not seem to contribute to starch accumulation in *gpt1* GCs, we isolated *gpt1pgi* homozygous double mutant plants (Supplemental Figure S1) to assess the impact of loss of PGI in *gpt1* mutant on GC starch accumulation (Figure 3A). Overall, *gpt1pgi* GCs accumulated starch similarly to *gpt1* (Figure 2B) or *gpt1gpt2* (Figure 2D) mutants. However, while *pgi* single mutants had elevated amounts of starch during the night (Figure 1A), starch contents were essentially identical between WT and *gpt1pgi* GCs throughout the night (Figure 3A and Supplemental Table S1). Thus, overaccumulation of starch in *pgi* GCs during the night may result from activation of GPT1 in *pgi* mutant background.

Similarly to *pgi* single mutants, starch synthesis in *gpt1pgi* double mutants was affected early in the day, although *gpt1pgi* GCs displayed a more pronounced lag phase (Figures 3A and 1A for comparison), which resulted in negative starch synthesis rates between 2 and 3 h of light (*gpt1pgi*_2__–__3_, −0.11; *pgi*_2__–__3_, 0.13; WT_2__–__3_, 1.27; Supplemental Table S1). Starch contents remained then lower compared with WT for the remainder of the day (Figure 3A), even when GC starch accumulation in *gpt1pgi* increased markedly between 3 and 9 h, showing elevated starch synthesis rates compared with WT and the single mutants (Figure 3A; WT_3__–__6_, 0.65; *pgi*_3__–__6_, 0.46; *gpt1*_3__–__6_, 0.44; *gpt1pgi*_3__–__6_, 2.00; WT_6__–__9_, 0.20; *pgi*_6__–__9_, 1.18; *gpt1*_6__–__9_, 0.59; *gpt1pgi*_6__–__9_, 0.79; Supplemental Table S1).

The lack of early starch synthesis (e.g. between 2 and 3 h) in combination with the reduced starch contents throughout the day indicate that both PGI and GPT1 are required for proper GC starch synthesis. Residual accumulation of starch in *gpt1pgi* mutants could be due to transcriptional upregulation of *GPT2* in this genetic background. However, we did not observe elevated amounts of *GPT2* transcripts in GC-enriched epidermal peels of *gpt1pgi* relative to WT at the EoN (Supplemental Figure S2).

### Triple *gpt1gpt2pgi* mutants are nearly devoid of GC starch

To explore if GPT2 or PGI partially contributed to GC starch accumulation in *gpt1pgi* or *gpt1gpt2* double mutants, respectively, we generated plants lacking all three enzymes. Due to the leaky *pgi* mutation, as described above, we transcriptionally downregulated *PGI* in *gpt1gpt2* mutant background, using artificial microRNA-based silencing (MIGS; Schwab et al., 2010). Given that PGI has a key role in mesophyll starch metabolism (Yu et al., 2000), and dramatically affects whole plant growth (Supplemental Figure S3), we expressed the corresponding amiRNA-*PGI* construct under the control of the GC-specific promoter of potassium influx channel (*KST1*) gene from potato (Kelly et al., 2013). Quantitative reverse transcription PCR (RT-qPCR) analyses on GC-enriched epidermal peels and intact rosette leaves confirmed GC-specific downregulation of *PGI* in *gpt1gpt2* mutant background for two independent lines, amiR-*PGI* #1 and #2 (Figure 3B). *PGI* transcripts in GCs were reduced by approximately 30% and 40%, respectively, while the expression in leaves was comparable to that of WT, at least for amiR-*PGI* #1 (Figure 3B).

GC starch contents were severely reduced at the EoN in the amiR-*PGI* #1 and #2 silencing lines compared with WT (Figure 3, C and D) and the respective single *pgi*, *gpt1*, *gpt2* mutants and *gpt1pgi*, *gpt1gpt2* double mutants (Figures 1A, 3A, and 2, B and D). Moreover, amiR-*PGI* #1 and #2 silencing lines accumulated GC starch at a much slower pace compared with WT and the mutants between 2 and 9 h of light, after which starch surprisingly started to accumulate similarly to WT, if not even at a higher rate (Figure 3, C and D) (WT_6__–__9_; 0.18; WT_9__–__12_; 0.27; amiRNA-*PGI* #1_6__–__9_; 0.23; amiRNA-*PGI* #1_9__–__12_; 0.42; amiRNA-*PGI* #2_6__–__9_; −0.10; amiRNA-*PGI* #2_9__–__12_; 0.85; Supplemental Table S3).

These data suggest that G6P derived from plastidial PGI-mediated conversion of F6P or imported through GPT2, and particularly GPT1, is the main substrate for GC starch synthesis up to approximately 9 h into the day. Hence, GC starch accumulation in *gpt1gpt2* and *gpt1pgi* double mutants likely resulted from activity of PGI and GPT2, respectively.

### GC-specific silencing of *PGM* leads to impaired starch accumulation until 9 h into the day

**Figure 4:**
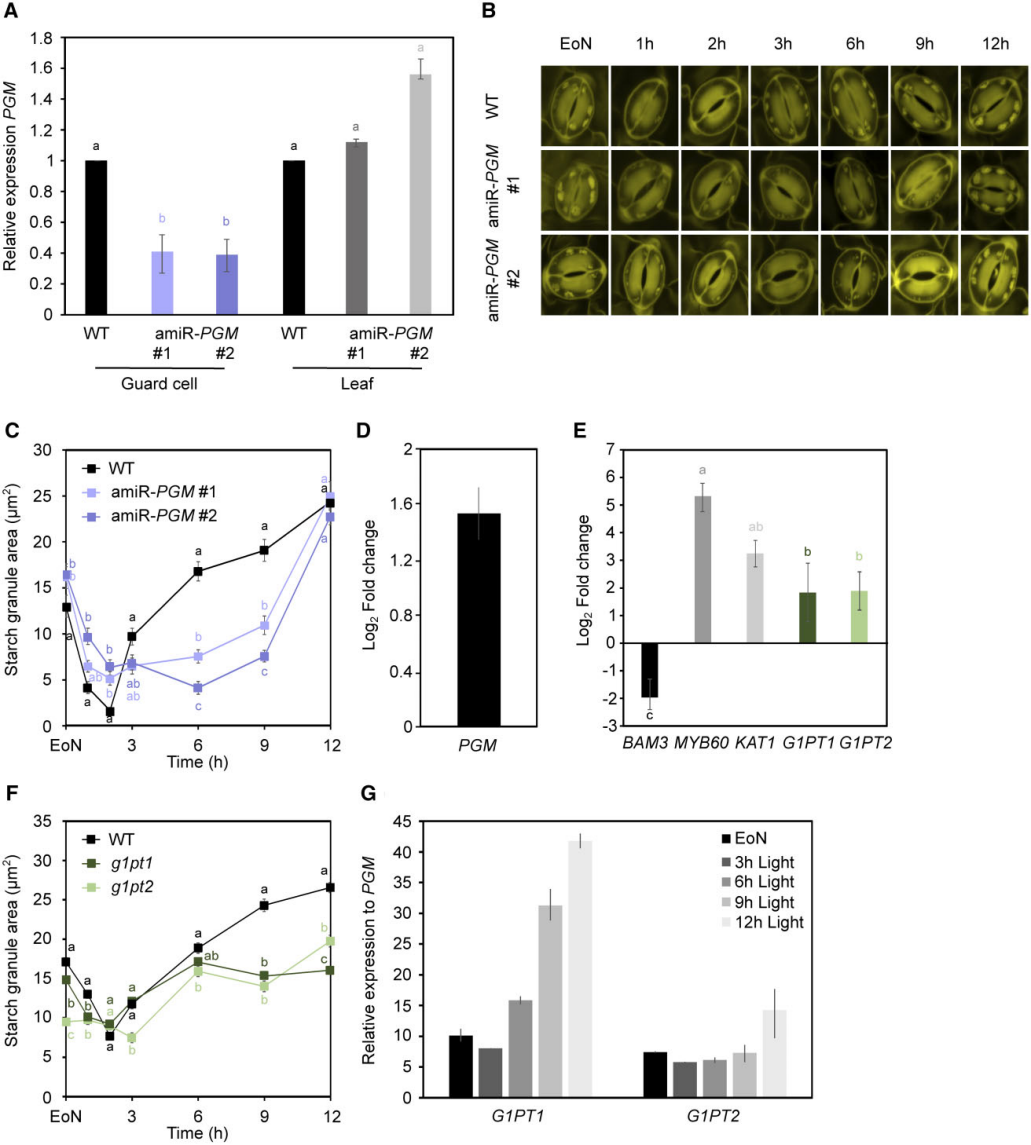
GC starch contents in *PGM* silencing lines in *gpt1gpt2* backgrounds. A, *PGM* gene expression in GC-enriched epidermal peels of artificial microRNA-induced silencing lines of *PGM* in WT genetic background (amiRNA-*PGM*) relative to WT GC-enriched epidermal peels and in intact rosette leaves of amiRNA-*PGM* lines relative to WT intact rosette leaves at EoN. Data from two experiments are shown; means ± fold change range; *n* = 6. B, Representative confocal images of propidium iodide-stained starch granules in GCs of intact leaves of WT and amiRNA-*PGM* plants over the 12 h light period. Scale bar = 10 µm. C, Starch dynamics in GCs of intact leaves of WT and amiRNA-*PGM* plants over the 12-h light period. Data from three experiments are shown; means ± sem; *n* = 120 individual GCs per genotype and time point. D, *PGM* gene expression in WT GC-enriched epidermal peels at 6 h relative to 3 h into the day. E, *G1PT1* and *G1PT2* gene expression in WT GC-enriched epidermal peels relative to WT intact rosette leaves at the EoN. *KAT1* and *MYB60* were used as markers for GC-specific expression, while *BAM3* was used as a leaf-specific marker. Data from two independent experiments are shown; means ± fold change range; *n* = 6. F, Starch dynamics in GCs of intact leaves of WT, *g1pt1*, and *g1pt2* plants over the 12-h light period. Data from three experiments are shown; means ± sem; *n* = 120 individual GCs per genotype and time point. G, *G1PT1* and *G1PT2* gene expression relative to *PGM* gene expression over the 12 h light period in WT GC-enriched epidermal peels. Means ± fold change range; *n* = 3. A, D, E, and G, *ACT2* was used as a housekeeping gene for normalization. For details about fold change and error calculations, refer to “Materials and methods.” Primer sequences and efficiencies are given in Supplemental Table S7. Letters indicate significant statistical difference between genes for *P* < 0.05 determined by one-way ANOVA with post hoc Tukey’s test. C and F, Plants were illuminated with 150 µmol m^−2^ s^−1^ of white light. Letters indicate significant statistical difference between genotypes for the given time point for *P* < 0.05 determined by one-way ANOVA with post hoc Tukey’s test. A–C and F, WT.

Starch formation in GCs of amiR-*PGI* silencing lines, which was particularly pronounced after 9 h of light (Figure 3D), suggests that (1) GCs have additional pathways for the synthesis of G6P and/or ii) in GC chloroplasts, conversion of G6P to G1P by PGM can be circumvented by uptake of cytosolic G1P. This would differ from metabolism in leaf chloroplasts, where PGM is required for starch synthesis and plant growth (Caspar et al., 1985; Paparelli et al., 2013). Arabidopsis plants lacking PGM, as in the EMS mutant *pgm1-1* (herein called *pgm*), are devoid of leaf starch (Caspar et al., 1985) and have severely impaired plant growth. To avoid pleiotropic effects on stomatal function, we silenced *PGM* specifically in GCs of WT plants by expressing amiRNA-*PGM* construct under the control of the GC-specific promoter *KST1*. We isolated two independent *PGM* silencing lines, amiR-*PGM* #1 and #2 (Figure 4A)*.* In both lines, *PGM* transcripts in GCs were reduced by ∼60%, whereas *PGM* expression in the leaves was comparable to WT (Figure 4A).

Starch was still present in GCs of both amiR-*PGM* lines (Figure 4, B and C). Starch levels were surprisingly elevated at the EoN in the amiR-*PGM* GCs compared with WT (Figure 4, B and C). Upon illumination, starch was degraded in all three genotypes, but at a markedly lower rate in the silencing lines (WT_0.1_,−0.59; WT_1__–__2_, −0.53; amiR-*PGM* #1_0__–__1_, −0.57; amiR-*PGM* #1_1__–__2_, −0.15; amiR-*PGM* #2_0__–__1_, −0.44; amiR-*PGM* #2_1__–__2_, −0.30; Supplemental Table S4), resulting in higher amounts of starch after 2 h of light (Figure 4, B and C). Between 2 and 9 h, WT GCs progressively accumulated starch, as expected, while starch contents remained unaltered in GCs of amiR-*PGM* #2 line and only mildly increased in amiR-*PGM* # 1 line (Figure 4, B and C;Supplemental Table S4). After 9 h into the day, however, starch synthesis rates greatly increased in both silencing lines (WT_9__–__12_, 0.27; amiR-*PGM* # 1_9__–__12_, 1.44; amiR-*PGM* # 2_9__–__12_, 1.68; Supplemental Table S5), reaching similar starch levels as the WT by the EoD (Figure 4, B and C).

Given that silencing of *PGM* was not complete (Figure 4A), we cannot exclude that starch accumulation in the silencing lines was due to residual PGM activity. That said, if the extent of *PGM* downregulation was the only factor affecting GC starch dynamics, we would expect a constitutive reduction of GC starch contents in amiR-*PGM* lines. This was not the case. The unique pattern of GC starch loss and formation in the amiR-*PGM* lines (Figure 4, B and C) rather points toward a role for PGM in GC starch metabolism at specific time of day, particularly between 2 and 9 h of light. Consistent with this idea, we found that *PGM* gene expression was ∼3.5-fold upregulated in GCs at 6 h compared with 3 h into the day (Figure 4D), further supporting the GC starch data. Altogether, these data suggest that, unlike MCs, GCs make starch using G1P either produced within the chloroplast through PGM or directly imported from the cytosol, depending on the time of day.

### Mutation of G1PT1 and G1PT2 transporters impairs GC starch accumulation toward the EoD

Uptake of G1P into Arabidopsis MC protoplasts and isolated chloroplasts was previously reported, including the rapid incorporation of imported G1P into starch (Fettke et al., 2011). In a recent follow-up study, the same authors identified two genes encoding UDP-rhamnose/UDP-galactose transporters, which can translocate G1P; At1g34020 (herein called G1PT1) and At4g09810 (herein called G1PT2). Arabidopsis *g1pt1g1pt2* double mutant plants showed reduced transport of G1P and mild alterations in leaf starch and sugar metabolism (Malinova et al., 2019).

Interestingly, both *G1PT* genes were highly expressed in GCs (Figure 4E). We, therefore, hypothesized that G1P may be synthesized in the cytosol through the cytosolic isoforms of PGM (PGM2 and PGM3; Egli et al., 2010) and subsequently translocated across the GC chloroplast membrane via the G1PT transporters. To assess the contribution of G1PTs to GC starch metabolism, we obtained homozygous T-DNA mutant lines of *G1PT1* (GABI_099E03) and *G1PT2* (SALK_123601) and analyzed stomatal starch dynamics throughout the 12 h light period (Figure 4F). Up until 6 h into the day, WT and *g1pt1* mutant displayed comparable changes in GC starch contents, after which *g1pt1* mutant remarkably stopped accumulating starch, reaching the EoD with considerably less starch than WT (Figure 4F). GC starch dynamics in *g1pt2* mutant were similar to those of *g1pt1*, although *g1pt2* mutants had considerably less starch at the EoN compared with both WT and *g1pt1* mutant, and starch levels remained unaltered during the first 3 h of light (Figure 4F). Starch synthesis in *g1pt2* GCs between 3–6 h and 9–12 h of light then occurred at increased rates compared with WT and *g1pt1* GCs (WT_3__–__6_, 0.61; WT_9__–__12_, 0.09; *g1pt1*_3__–__6_, 0.41; *g1pt1*_9__–__12_, 0.05; *g1pt2*_3__–__6_, 1.12; *g1pt2*_9__–__12_, 0.41; Supplemental Table S5), reaching at the EoD comparable levels of starch to that of *g1pt1* mutant (Figure 4F).

The GC starch phenotype of *g1pt* mutants markedly differed from that of amiR-*PGM* silencing lines, particularly between 6 and 12 of light, during which GC starch dynamics showed opposite trends (Figure 4, F versus C). To assess whether G1PT transporters play a complementary role to PGM in providing G1P for GC starch synthesis, we next compared *G1PTs* transcript levels with that of *PGM* in WT GC-enriched epidermal peels harvested throughout the 12 h light period (Figure 4G). Both *G1PTs* genes were expressed at higher levels compared with *PGM* at all investigated time points (Figure 4G). The expression of *G1PT1* and *G1PT2* was, respectively, ∼10- and 8-fold higher compared with *PGM* at the EoN (Figure 4G), coinciding with the elevated amounts of GC starch in the *PGM* silencing lines (Figure 4, B and C). Starting from 6 h of light, *G1PT1* gene expression was substantially higher than *PGM*, showing up to ∼43-fold upregulation at the EoD (Figure 4G). This again matched the formation of starch between 9 and 12  h of light in the two PGM silencing lines (Figure 4, B and C). Compared with *G1PT1*, the expression of *G1PT2* relative to *PGM* was not markedly different, but overall it followed a similar pattern of that of *G1PT1*, showing ∼14-fold upregulation at EoD (Figure 4G). Altogether, our data suggest that G1PT activity is crucial for starch synthesis in GCs, particularly between 6 and 12 h of light, when PGM seems to play a minor role.

Besides the cytosolic formation of G1P and the subsequent uptake into chloroplasts, heterotrophic cells can also form starch from direct transfer of G1P onto growing starch glucan chains with the help of PHS1. This pathway was shown to operate in heterotrophic storage tissues of potato, rice, and wheat (Satoh et al., 2008; Tickle et al., 2009; Fettke et al., 2010). PHS1 catalyzes the reversible phosphorolytic cleavage of α-1,4-glycosidic bonds of starch (Zeeman et al., 2004). *PHS1* transcripts were approximately four-fold upregulated in GCs compared with leaves (Supplemental Figure S4a and Supplemental Table S1). However, loss of PHS1 had no major impact on GC starch dynamics during the day, as demonstrated by the fact that T-DNA *phs1* single mutants (GABI_257A06) accumulated starch similarly to WT (Supplemental Figure S4b and Supplemental Table S6). Hence, PHS1 does not seem to be involved in daytime starch synthesis in GCs.

### Simultaneous loss of APL3 and APL4 large subunits of AGPase impairs GC starch accumulation throughout the day

The conversion of G1P into ADPGlc by AGPase represents a bottleneck in the starch biosynthetic pathway (Stitt and Zeeman, 2012). AGPase is a highly regulated enzyme, composed of two small subunits (APS1-2) and two large subunits (APL1-4). The leaf enzyme consists of two catalytic APS1 subunits and two regulatory APL1 subunits. APL3 and APL4 regulatory subunits are preferentially expressed in sink tissues, while APL2 is generally expressed at negligible levels (Crevillén et al., 2003, 2005). There is evidence that the combination of the regulatory large subunits influences the catalytic activity of AGPase (Crevillén et al., 2003, 2005). The composition of the AGPase enzyme in GCs is unknown.

**Figure 5:**
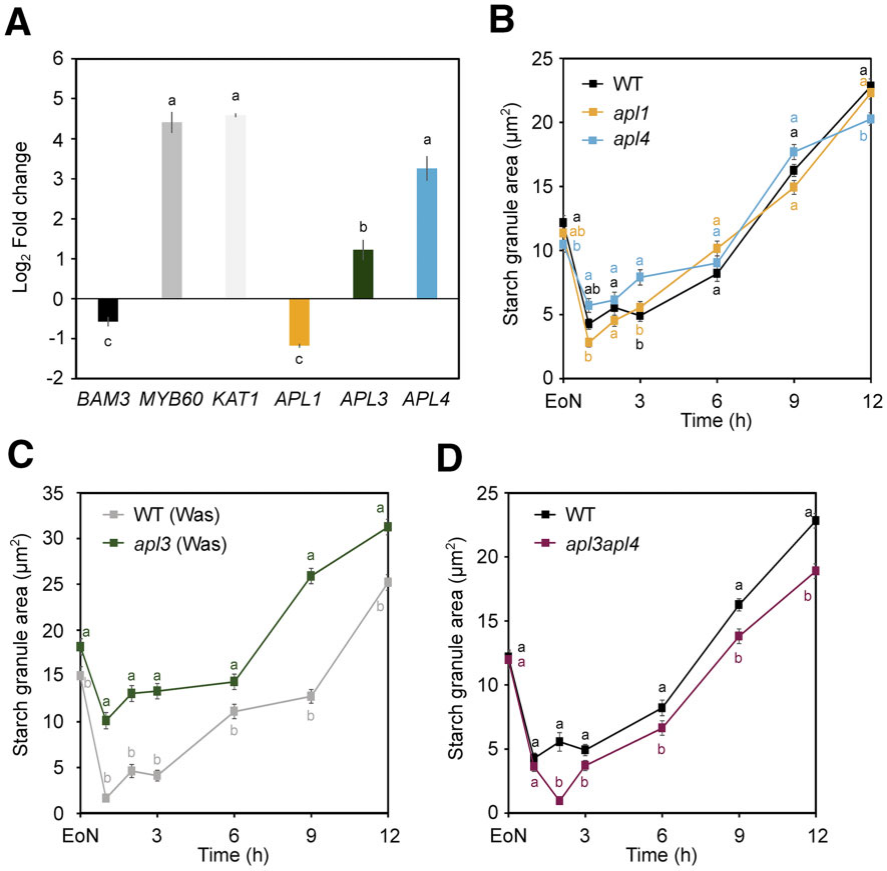
GC gene expression of *APLs* and GC starch contents in *apl* mutants. A, *APL1*, *APL3*, and *APL4* gene expression in WT GC-enriched epidermal peels relative to WT intact rosette leaves at EoN. *KAT1* and *MYB60* were used as markers for GC-specific expression, while *BAM3* was used as a leaf-specific marker. Data from two experiments are shown; means ± fold change range; *n* = 6. *ACT2* was used as a housekeeping gene for normalization. For details about fold change and error calculations, refer to “Materials and methods.” Primer sequences and efficiencies are given in Supplemental Table S7. Letters indicate significant statistical difference between genes for *P* < 0.05 determined by one-way ANOVA with post hoc Tukey’s test. Starch dynamics in GCs of intact leaves of (B) WT and *apl1*, *apl4*, (C) Was and *apl3*, (D) WT and *apl3apl4* plants. Data from four experiments are shown; means ± sem; *n* = 160 individual GCs per genotype and time point. Plants were illuminated with 150 µmol m^−2^ s^−1^ of white light. WT data are the same as in (B). Letters indicate significant statistical difference between genotypes for the given time point for *P* < 0.05 determined by one-way ANOVA with post hoc Tukey’s test. B–D, WT.

We examined *APL* gene expression in WT GC-enriched epidermal peels relative to intact rosette leaves at the EoN (Figure 5A). As previously reported, *APL1* was preferentially expressed in leaf tissues similarly to the leaf marker gene *β-amylase 3* (*BAM3*; Figure 5A; Crevillén et al., 2003). *APL3* and *APL4* on the other hand were both highly expressed in GCs compared with leaves, with *APL4* showing a more pronounced preferential GC expression compared with *APL3* (Figure 5A).

Based on the promising RT-qPCR results, we quantified stomatal starch contents in the EMS mutant *apl1* (Lin *et al.*, 1988) and the T-DNA insertion mutants *apl3* (Was background; FLAG_458A07) and *apl4* (SALK_108632) throughout the 12 h light period. As expected, GC starch dynamics were indistinguishable between WT and the *apl1* mutant (Figure 5B and Supplemental Table S6). Even if *APL4* gene was upregulated in GCs, *apl4* single mutants accumulated starch similarly to WT (Figure 5B and Supplemental Table S6). By contrast, starch contents were constitutively elevated in *apl3* GCs compared with the corresponding Was WT control, and starch synthesis was impaired specifically between 2 and 6 h of light (Figure 5C), indicating a deregulation of the GC AGPase enzyme in the absence of APL3. Furthermore, WT and *apl3* mutant in the Was background contained slightly elevated levels of both GC starch (Figure 5C) and leaf starch (Supplemental Figure S5) compared with WT of Col-0 background. Starch contents in the leaves of *apl3* single mutants were further elevated compared with Was control plants, supporting the idea of a deregulated enzyme (Supplemental Figure S5).

To assess functional interaction between APL3 and APL4 subunits in GC starch metabolism, we generated the *apl3apl4* double mutant through initial backcrossing of the *apl3* mutation into Col-0 WT background, followed by classical mutant crossing. Combined loss of APL3 and APL4 resulted in overall reduced GC starch amounts (Figure 5D and Supplemental Table S6). WT and *apl3apl4* GCs contained comparable amounts of starch at the EoN (Figure 5D), which was degraded similarly upon light exposure (Figure 5D and Supplemental Table S6). However, while WT GCs gradually increased starch contents from 1 h into the day onward, GCs of the double mutant displayed a net decrease of starch between 1 and 2 h (Figure 5D; WT_1__–__2_, 0.30; *apl3apl4*_1__–__2_, −0.74; Supplemental Table S6). Thereafter, starch contents in the *apl3apl4* mutant remained at a lower level until the EoD compared with WT (Figure 5D and Supplemental Table S6). We conclude that APL3 and APL4 are the major large subunits of GC AGPase enzyme. Furthermore, APL3 and APL4 seem to have partially redundant functions in GC starch accumulation.

### Mutation of *BT1* has no impact on GC starch accumulation

Unaffected levels of ADPGlc in mutants of PGM and AGPase prompted to rethinking the classical model of starch biosynthesis (Muñoz et al., 2005). Several reports suggested that ADPGlc can be generated in the cytosol through the activity of Suc synthases (SUS) and subsequently translocated across the chloroplast membrane (Baroja-Fernández et al., 2003; Muñoz et al., 2005, 2006). Interestingly, transcriptomic studies revealed high abundance of SUS3 in GCs (Bates et al., 2012). Moreover, plastids of maize endosperm were shown to be able to import cytosolic ADPGlc (Shannon et al., 1998) via the plastidic ADPGlc transporter BT1 (Kirchberger et al., 2007). Similar observations were made for rice endosperm (Li et al., 2017). The Arabidopsis genome encodes a *BT1* gene homolog (*BT1*), which is structurally similar to that of maize. BT1 localizes to the plastidial membrane and was described to mediate AMP, ADP, and ATP transport into the chloroplast. However, in vitro experiments showed that BT1 does not accept ADPGlc as a substrate (Kirchberger et al., 2008). Here, we tested whether BT1 contributes to starch metabolism in GCs, potentially providing a precursor for starch synthesis between 6 and 9 h into the day.

*BT1* gene expression was almost four-fold higher in WT GCs relative to intact leaves (Supplemental Figure S6a). However, GC starch dynamics in T-DNA BT1 single mutants (SALK_026943) were similar to those of WT throughout the 12 h light phase (Supplemental Figure S6b), except for time point 12 h, in which BT1 GCs contained significantly more starch (Supplemental Figure S6b). Hence, we conclude that BT1 is not required for stomatal starch accumulation. We suggest that BT1 might have a different function than importing ADPGlc, for example, it may facilitate the exchange of ATP, ADP, and AMP, as it was described for other plant tissues (Kirchberger et al., 2008).

## Discussion

### PGI and GPT1 provide G6P precursor for diurnal GC starch biosynthesis in a temporally coordinated manner

In the leaf mesophyll, starch synthesis strictly depends on PGI enzyme, which produces G6P precursor from the CBB cycle intermediate F6P (Yu et al., 2000). Through extensive single and multiple mutant analyses (Figures 1–3), we provide evidence that in GC chloroplasts, G6P for starch synthesis not only originates from the PGI-mediated reaction, but it can also be imported from the cytosol through GPTs, particularly GPT1. Both PGI and GPT1 are required for proper GC starch synthesis, as demonstrated by altered starch accumulation profiles in the corresponding single mutants (Figures 1A and 2B). However, while the PGI reaction is essential during the early phase of GC starch synthesis (i.e. 2–3 h of light, Figure 1A), import of G6P via GPT1 plays a critical role for starch accumulation later during the day (Figure 2B). This observation is further supported by gene expression data showing that *GPT1* gene was upregulated at 6 h of light relative to 3 h, while *PGI* was not (Figure 2C).

These findings have important implications. First, they suggest that autonomous CO_2_ fixation in GCs does occur to levels which contribute F6P for starch synthesis. Hence, CBB cycle is functional in GCs (Lawson and Matthews, 2020), despite some earlier studies on species such as broad bean (*Vicia faba*) or pea reported otherwise (Hedrich et al., 1985; Outlaw, 1989; Reckmann et al., 1990). Our conclusion is in line with a very recent study showing that the enzymes for phototropic CO_2_ fixation are present in small amounts in Arabidopsis GCs and GC photosynthesis at least partly contributes to starch synthesis in GC chloroplasts (Lim et al., 2022).

That said, the observed accumulation of starch in *pgi* GCs, which is in contrast with the situation in MCs, demonstrates that GCs also have features of heterotrophic organs, which metabolism depends on imported sugars. In line with this idea, we previously showed isolated GCs accumulate substantially less starch than GCs of intact leaves (Flütsch et al., 2020a). Furthermore, mesophyll-derived Glc imported to GCs via plasma membrane monosaccharide-H^+^ symporters SUGAR TRANSPORT PROTEIN 1 and 4 (STP1 and STP4) represents the major carbon source for GC starch biosynthesis (Flütsch et al., 2020b). Imported Glc may be phosphorylated by cytosolic hexokinases prior to translocation across the plastidial envelope by GPTs.

Second, the GC starch phenotype of the analyzed *pgi* and *gpt* mutants (Figures 1A and 2B) further suggests that PGI and GPT1 are active at different times of the day, likely as a result of differential regulation of diurnal gene expression (Figure 2C). Various factors might affect *PGI* and *GPT1* expression in GCs, for instance signals from light receptors at the plasma membrane, redox state of cellular compartments, or concentration of metabolites, predominantly sugars (Häusler et al., 2014). In such a scenario, it is plausible to imagine that in the morning, when stomata are fully open, GC photosynthesis is more active and provides sufficient F6P amounts to fuel the PGI reaction. In the afternoon, when stomata tend to close as the plant becomes carbon-saturated (Jakobson et al., 2016; Yaaran et al., 2019), the combination of reduced GC photosynthesis, along with the need of removing organic metabolites previously stored in the vacuole to promote stomatal closure, may activate GPT1 activity. This hypothesis is supported by previous studies showing that starch biosynthesis in GCs is involved in high CO_2_-induced stomatal closing, where starch would serve as a sink for metabolites previously accumulated within GCs, which need to be removed to reduce cell turgor (Penfield et al., 2012; Azoulay-Shemer et al., 2016, 2018).

Our analyses also suggest that starch accumulation in darkness does not depend on either PGI or GPT1, as starch levels in GCs of *gpt1pgi* double mutants were comparable to that of WT during the entire night (Figure 3A). We cannot exclude that GPT2 may compensate for the loss of PGI and GPT1, although gene expression analyses did not support such hypothesis (Figure 1C and Supplemental Figure S2), which might have been related to the sampling time point at the EoN. *GPT2* expression was indeed reported to be repressed in the dark (Kunz et al., 2010).

While our work establishes a crucial role for PGI and GPT1 in GC starch biosynthesis, residual accumulation of starch in *amiR-PGI* silencing lines, in which *PGI* was downregulated in *gpt1gpt2* background (Figure 3, C and D), indicates that G6P in GC chloroplasts can originate yet through alternative pathways, which may circumvent both PGI and GPTs. A potential way of G6P formation in chloroplasts involves the import of cytosolic Glc via the plastid-localized Glc transporter (pGlcT) and/or the recently characterized plastidic sugar transporter (pSuT) (Patzke et al., 2019). Once in the chloroplast, Glc can be phosphorylated to G6P by the plastidial HXK3 (Weber et al., 2000; Karve et al., 2008). However, both pGlcT and pSuT reactions seem to promote export of Glc from the chloroplast rather than uptake of cytosolic Glc (Weber et al., 2000; Cho et al., 2011; Patzke et al., 2019), and the respective mutants have WT-like levels of starch, at least in leaves (Cho et al., 2011; Patzke et al., 2019). Although it cannot be excluded that pGlcT and/or pSuT might catalyze—under selected conditions—sugar import to the chloroplast, it seems unlikely that GCs make starch using cytosolic Glc, but additional research would be needed to exclude this route of G6P provision.

### PGI and GPTs can partially complement each other function in GCs

Previous studies reported that constitutive expression of either GPTs rescued the leaf starch deficient phenotype of *pgi* mutant, indicating GPTs can compensate for the loss of PGI (Kammerer et al., 1998; Niewiadomski et al., 2005; Kunz et al., 2010). Our work suggests indeed that a certain level of reciprocal functional complementation between PGI and GPTs also occurs in GCs.

For instance, despite *GPT2* was highly expressed in GCs relative to leaves (Figure 2A), loss of GPT2 alone or in the *gpt1* mutant background had no major impact on GC starch accumulation (Figure 2, B and D). However, GPT2 was specifically activated in GCs in the absence of both GPT1 and PGI, as in the *amiR-PGI* silencing lines, which showed more severe reductions in GC starch accumulation than either of the single *pgi*, *gpt1* mutants or *gpt1pgi*, *gpt1gpt2* double mutants (Figures 3, C and D, 1A, 3A, and 2, B and D). While we cannot explain the activation of GPT2 by increased gene transcription (Supplemental Figure S2), it should be noted that responses at the mRNA level do not always reflect changes in protein amounts or enzyme activity. Recent work revealed that GPT proteins, despite having highly conserved catalytic and substrate binding sites, diverge substantially in their N-terminal domains (Baune et al., 2020), suggesting posttranslational modifications may play a role in regulating GPT activity.

Additional evidence of functional compensation between PGI and GPTs is provided by the GC starch phenotypes of *pgi* and *gpt1pgi* mutants. While *pgi* showed elevated starch levels during the night (Figure 1A), *gpt1pgi* double mutants had starch amounts comparable to WT (Figure 3A), suggesting that nighttime starch overaccumulation in *pgi* GCs resulted from GPT1 activation. Lastly, the mild GC starch phenotype of *gpt1gpt2* (Figure 2D), besides indicating that GPTs do not have redundant functions in GCs, suggests that PGI compensated for the loss of both GPTs.

We suggest that the functional interaction between PGI and GPTs in GCs is a unique feature of GC starch metabolism, likely to compensate for the limited photosynthetic capacity of GCs compared with leaves.

### PGM and G1PTs provide G1P precursor for diurnal GC starch biosynthesis in a temporally coordinated manner

The second critical step of the starch biosynthesis pathway is the generation of G1P as a substrate for the AGPase. Our work unequivocally demonstrates that, similarly to G6P, there are at least two sources of G1P for GC chloroplasts: (1) the PGM-catalyzed conversion of G6P within the chloroplast stroma and (2) G1P imported from the cytosol via G1PTs.

In MCs, the PGM reaction is absolutely required for starch metabolism, as *pgm* mutants suffer from severely reduced growth and disturbed carbohydrate metabolism (Caspar et al., 1985). Previous studies reported *pgm* GCs to be devoid of starch (Lasceve et al., 1997; Horrer et al., 2016). However, starch granules were only visualized at the beginning of the day (0–3 h of light) (Lasceve et al., 1997; Horrer et al., 2016). Here, we thoroughly investigated GC starch dynamics in two independent amiRNA-*PGM* lines, where *PGM* was silenced specifically in GCs to avoid potential pleiotropic effects deriving from the well-known diurnal overaccumulation of sugars in the leaves of *pgm* (Caspar et al., 1985). We revealed that, unlike MCs, provision of G1P by PGM is essential for starch biosynthesis in GCs only over a very specific time window, that is between 2 and 6 h of light, when plants are grown in a 12-h/12-h light/dark photoperiod (Figure 4, B and C).

The unexpected accumulation of starch in GCs of amiRNA-*PGM* lines starting from 6 h of light (Figure 4C) can be explained by the activity of two G1P transporters, G1PT1 and G1PT2. Almost ten years ago, it was demonstrated that both MC protoplasts and isolated chloroplasts have the capacity to import G1P and metabolize it into starch (Fettke et al., 2011). More recently, Malinova et al. (2019) identified two UDP-rhamnose/UDP-galactose transporters, G1PT1 and G1PT2, which are able to transport G1P (Rautengarten et al., 2011; Malinova et al., 2019). Using transient expression in Arabidopsis mesophyll protoplasts, it was revealed that both transporters localize at the plasma membrane (Malinova et al., 2019). However, earlier reports along with the examination of N-terminal targeting peptides suggest that G1PT2 is targeted to the plastid, whereas for G1PT1 no such peptide was identified (Knappe et al., 2003; ChloroP 1.1: http://www.cbs.dtu.dk/services/ChloroP/). Interestingly, in Supplemental Figure S1C of Malinova et al. (2019), showing the subcellular localization in transiently transformed protoplasts, a clear chloroplast signal is visible for G1PT1. Therefore, the localization of these two transporters is not yet fully resolved.

Regardless, we found that both *G1PT* genes were highly expressed in GCs (Figure 4E). Even more intriguingly, both genes, particularly *G1PT1*, were upregulated during the second half of the day in comparison to PGM (Figure 4G). Consistent with the gene expression profile, the corresponding *g1pt* single mutants (g*1pt1* and *g1pt2*) failed to accumulate starch in GCs specifically between 6 and 12 h of light (Figure 4F), in coincidence with the peak of starch synthesis observed in amiRNA-*PGM* lines (Figure 4C). Hence, the PGM reaction is not the only source of G1P for GCs, again suggesting GC starch biosynthesis has unique features of both autotrophic and heterotrophic cells. Similar to PGI and GPTs, PGM and G1PTs activity in GCs seems to be temporally coordinated, likely to compensate for limited autonomous photosynthesis of this cell type, and to contribute metabolite removal during stomatal closure.

### APL3 and APL4 are the major regulatory subunits of AGPase in GCs

The AGPase enzyme catalyzes the third and limiting step of starch synthesis and is regulated allosterically by the levels of 3-phosphoglyceric acid and inorganic phosphate in photosynthetic tissues, such as the spongy mesophyll (Preiss, 1982). Previous biochemical experiments have shown that the activity of the heterotetrametric enzyme depends on the combination of the small catalytic subunit APS1 with the large subunits APL1–APL4. In Arabidopsis leaves, APS1/APL1 heterotetramer had the highest sensitivity toward the allosteric effectors, while heterotetramers composed of APS1 and APL2-APL4 responded only to large changes in effector concentrations (Crevillén et al., 2003). The regulatory APL subunits also influence AGPase substrate affinity, with APL1 conferring the highest affinity toward ATP and G1P (Crevillén et al., 2003). In line with these findings, *APL1* gene expression was shown to be highest in leaves, whereas *APL3* expression peaked in sink organs, such as inflorescences, fruits and roots (Crevillén et al., 2005). Microarray analyses further indicated *APL4* is the most abundant AGPase large subunit in GCs of Arabidopsis (Leonhardt et al., 2004).

In this study, we demonstrate that subunit composition of the GC AGPase enzyme differs from that of MCs. First, we corroborate earlier results showing that *APL3* and, to a higher extent, *APL4* were preferentially expressed in GCs relative to leaves, whereas *APL1* transcripts were highly abundant in leaf tissues (Figure 5A;Leonhardt et al., 2004; Crevillén et al., 2005). Second, we show that mutation of *APL1* had no impact on GC starch turnover (Figure 5B), while simultaneous loss of APL3 and APL4 in the *apl3apl4* double mutant resulted in overall reduced GC starch amounts throughout the 12 h light phase, suggesting APL3/APL4 are the main large subunits of the GC AGPase enzyme (Figure 5D;Supplemental Table S6). However, while *apl4* single mutants had WT-like GC starch accumulation profiles (Figure 5B), *apl3* single mutants showed altered GC starch levels, particularly between 2 and 6 h of light (Figure 5C). To our surprise, *APL3* mutation led to elevated, not reduced, starch amounts, both in GCs and in MCs (Figure 5C and Supplemental Figure S4).

The GC starch phenotypes of *apl3*, *apl4*, and *apl3apl4* mutants suggest an intricate functional interaction between APL subunits in GCs, which may also depend on the genetic background. The increased accumulation of starch in *apl3* mutants might result from overexpression of either of the remaining APL subunits, leading to functional complementation. Alternatively, the function of APL3 in Arabidopsis Was accession may differ from that in Col-0. However, also in the case of the *apl3apl4* double mutant, the remaining GC starch accumulation might be explained by an upregulation of APL1 or APL2, partially complementing for the absence of APL3 and APL4. It would be worth assessing the expression of *APL1* and *APL2* genes in *apl3apl4* GCs in future experimental work.

We also lack information about the regulation of AGPase activity in GCs. It was previously reported that sugars can transcriptionally induce both *APL3* and *APL4*, but not *APL1* or *APL2* (Crevillén et al., 2005). Thus, the activity of AGPase in sink tissues could be responding to sugar availability. This would be particularly relevant in GCs, where we already know sugars play a critical role in coordinating GC and MC metabolism to fulfill the need of the plant (Flütsch and Santelia, 2021).

## Materials and methods

### Plant material and growth conditions

All experiments were performed with non-flowering, four-week-old Arabidopsis (*A.* *thaliana*) plants in the accession Columbia (Col-0 = WT) background. Transfer DNA (T-DNA) insertion lines SALK_026943 (*BT1*), GABIKAT_454H06 (*gpt2*), GABI_099E03 (*g1pt1*) and SALK_123601 (*g1pt2*) and GABI_257A06 (*phs1*) were obtained from Nottingham Arabidopsis Stock Centre (NASC). SALK_108632 (*apl4-3*) line was provided by Samuel Zeeman (ETH Zürich, CH). Alison Smith (John Innes Centre, UK) provided FLAG_458A07 (*apl3-1*) in the accession Wassilewskija (Was) background. Ethyl methanesulfonate (EMS) mutants *apl1* (*adg2;*Lin et al., 1988), *pgm1-1* (Caspar et al., 1985), and *pgi1-1* (Yu et al., 2000) were described previously.

Mutations affecting *GPT1* were previously described to be embryo lethal. However, viable, homozygous T-DNA lines for the GPT1 locus are available (*gpt1-3, gpt1-5*, and *gpt1-6*), which were characterized to have unaltered *GPT1* transcript amounts (Niewiadomski et al., 2005). A more recent detailed analysis of SALK_021762 (*gpt1-3* allele) found substantial reductions of *GPT1* transcripts in different flower organs along with reduced starch contents (Hedhly et al., 2016). Hence, in this study we used the *gpt1-3* allele obtained from NASC.

The *apl3-1apl4-3* double mutant was created firstly by backcrossing the *apl3-1* mutant into Col-0 WT to eliminate the Was background. Subsequent crosses using either Col-0 WT or backcrossed *apl3-1* mutant as pollen donor yielded heterozygous plants in the expected ratio. In each generation, heterozygous *apl3-1* mutant plants were selected by genotyping using the primers listed in Supplemental Table S7. Heterozygous *apl3-1* mutants of the fourth generation were crossed with homozygous *apl4-3* plants and double homozygous mutants selected by molecular genotyping using primers combination as listed in Supplemental Table S7.

The *gpt1pgi1-1* and *gpt1gpt2* double mutant plants were generated through standard crossing techniques and isolated by molecular genotyping (for primer sequences see Supplemental Table S7). Genotyping of the *pgi1-1* point mutation was done by sequencing of the PCR product obtained with the primers listed in Supplemental Table S7. An aliquot of 1  µL of purified genomic DNA was used in a PCR reaction, followed by column purification using the Wizard SV Gel and PCR Clean-Up System (Promega, Dübendorf, Zürich, CH). An aliquot of 2 µL of the purified PCR reaction (around 100 ng) were used for sequencing. Sequencing chromatograms were analyzed for single C to T substitution at base 834 (Supplemental Figure S7; Yu et al., 2000).

Plants were cultivated in soil in controlled-climate chambers (Fitoclima 1200, Aralab; ClimeCab 1400, Kälte3000; Klimaschrank from Kälte3000) under a 12-h/12-h light/dark photoperiod, with a temperature of 21°C/19°C day/night, a relative humidity of 45%/55% day/night and an irradiance of 150 µmol m^−2^ s^−1^ using LED tubes (Fitoclima 1200), LED panels (ClimeCab 1400) and halogen lamps (Klimaschrank).

### GC-specific gene silencing

For GC-specific gene silencing of *PGI* and *PGM*, sequences of pre-miRNAs were designed using the Web MicroRNA Designer tool (WMD3; http://wmd3.weigelworld.org/cgi-bin/webapp.cgi). The primer set listed in Supplemental Table S7 was used to incorporate the 21-bp amiRNA sequence into the MIR319a vector (Schwab et al., 2010). Subsequently, the amiRNA construct was subcloned into BJ36 (Moore et al., 1998) containing the GC-specific promoter *KST1* (Kelly et al., 2013). The resulting pSF10 (amiR-*PGM*) and pSF21 (amiR*-PGI*) constructs were transformed into Arabidopsis WT (pSF10) and *gpt1gpt2* (pSF21) backgrounds followed by selection of independent lines (Supplemental Table S8).

### GC starch quantification

GC starch was quantified at the indicated time points. Epidermal peels were manually obtained from leaf number 5 or 6. GC starch granules were fixed and stained as previously described (Flütsch et al., 2018). Subsequently, GC starch granules were visualized and imaged using a confocal laser-scanning microscope Leica TCS SP5 (Leica Microsystems) or Zeiss LSM 780 (Zeiss) using the following set up: Argon laser 5%; Objective 63×, glycerol; Excitation 488 nm; Detector HyD3; Emission filter 610–640 nm; Format 1,024 × 1,024 pixel; Zoom 6× (Flütsch et al., 2018). Starch granule area was measured using ImageJ version 1.48 (NIH USA, http://rsbweb.nih.gov/ij/). To avoid overlapping signals with starch granules from MCs, images of GC starch granules were acquired only from mesophyll-free parts of the epidermal peels. Four biological replicates were analyzed per genotype and time point for each experiment.

### Leaf and GC RNA isolation and RT-qPCR

To extract leaf RNA, three entire rosettes per genotype and time point (three biological replicates) were harvested at the indicated time points and frozen in liquid nitrogen.

To extract RNA from GC-enriched epidermal peels, the middle veins of 12 rosettes per genotype and time point (one biological replicate) were excised at the indicated time points and the remaining leaf material was blended in 100 mL ice-cold water using a kitchen blender (ProBlend Avance collection, Philips). Blended sample was filtered through a 200-µm nylon mesh (Sefar), and the remaining epidermal peels were dried, collected in a tube, and immediately frozen in liquid nitrogen.

Subsequently, the epidermal peels were ground to a fine powder using a tissue grinder (Mix Mill MM-301, Retsch). For each experiment, two or three biological replicates per genotype and time point were harvested. Two independent experiments were performed for each extraction (leaves and GC-enriched epidermal peels).

Total RNA was extracted from ≥30 mg of ground tissue using the SV Total RNA Isolation Kit (Promega) following the manufacturer’s instructions. RNA quality and quantity were analyzed with a NanoDrop ND-1000 spectrophotometer (Thermo Fisher Scientific, Waltham, MA, USA). A total of 1 µg of RNA was used for cDNA first-strand synthesis using the M-MLV Reverse Transcriptase RNase H Minus Point Mutant and oligo(dT)15 primer (Promega). Transcript levels were examined by RT-qPCR using the SYBR Green Master Mix (Applied Biosystems, Waltham, MA, USA) and the 7500 Fast Real-Time PCR System (Applied Biosystems). RT-qPCR was performed in triplicates. Transcript levels were calculated according to the comparative CT method (Livak and Schmittgen, 2001) and were normalized against the expression of the *Actin2* gene (*ACT2*; At3g18780). Error calculations were done according to Applied Biosystems guidelines (http://www3.appliedbiosystems.com/cms/groups/mcb_support/documents/generaldocuments/cms_042380.pdf). Primers and PCR efficiencies for RT-qPCR are listed in Supplemental Table S1.

### Statistical analysis

Statistical differences between genotypes and time points were determined by ANOVA with post hoc Tukey’s Honest Significant Difference test (*P*-value < 0.05). All data are indicated as means ± SEM.

## Data availability

All data supporting the findings of this study are available within the paper and within its supplementary materials published online.

## Accession numbers

Sequence data from this article can be found in the Arabidopsis Genome Initiative or GenBank/EMBL databases under the following accession numbers: At4g17090 (*BAM3*), At3g18780 (*ACT2*), At5g46240 (*KAT1*), At1g08810 (*MYB60*), At5g54800 (*GPT1*), At1t61800 (*GPT2*), At4g24620 (*PGI*), At5t51820 (*PGM*), At3t29320 (*PHS1*), At4g39210 (*APL1*), At4g39210 (*APL3*), At2g21590 (*APL4*), At4g32400 (*BT1*), At1g34020 (*G1PT1*), and At4g09810 (*G1PT2*).

## Supplemental data

The following materials are available in the online version of this article.

**Supplemental Figure S1.** Genotyping of *pgi* and *gpt1pgi* mutants.

**Supplemental Figure S2.** Gene expression of *GPT2* in *gpt1pgi* mutants.

**Supplemental Figure S3.** Growth retardation of *pgi* mutants.

**Supplemental Figure S4.** GC gene expression of *PHS1* and starch contents in *phs1* mutants.

**Supplemental Figure S5.** Leaf starch contents in *apl* mutants.

**Supplemental Figure S6.** GC gene expression of *BRITTLE1* and starch contents in *brittle1* mutants.

**Supplemental Table S1.** Starch synthesis rates of WT, *pgi* and *gpt* mutant GCs.

**Supplemental Table S2.** Starch synthesis rates of WT and *gpt1gpt2* GCs.

**Supplemental Table S3.** Starch synthesis rates of WT and *PGI* silencing lines GCs.

**Supplemental Table S4.** Starch synthesis rates of WT and *PGM* silencing lines GCs.

**Supplemental Table S5.** Starch synthesis rates of WT and *g1pt* mutant GCs.

**Supplemental Table S6.** Starch synthesis rates of WT, *phs1* and *apl* mutant GCs.

**Supplemental Table S7.** Oligonucleotides used in this study.

**Supplemental Table S8.** Plasmids generated in this study.

## Supplementary Material

kiac087_Supplementary_Data

## Notes

D.S. and S.F. conceptualized the research. S.F. performed the majority of the experiments. D.H. performed the crosses for the generation of the *gpt1pgi* and the *apl3apl4* double mutants, and contributed to data in Figures 1A, 2B, and 5, B–D, and Supplemental Figure S1, S3, and S5. S.F. analyzed all data. S.F. and D.S. wrote the manuscript with approval from D.H. D.S. agrees to serve as the author responsible for contact and ensures communication.

The author responsible for distribution of materials integral to the findings presented in this article in accordance with the policy described in the Instructions for Authors (https://academic.oup.com/plphys/pages/general-instructions) is: Diana Santelia (dsantelia@ethz.ch).
