# Supplementary material for: Starch biosynthesis in guard cells has features of both autotrophic and heterotrophic tissues
Source: Plant Physiol. 2022 Mar 3;189(2):541–56. doi: 10.1093/plphys/kiac087 (PMC9157084; doi:10.1093/plphys/kiac087)
Supplement: kiac087_Supplementary_Data [file kiac087_supplementary_data.pdf]

Supplemental Data Flütsch *et al.*

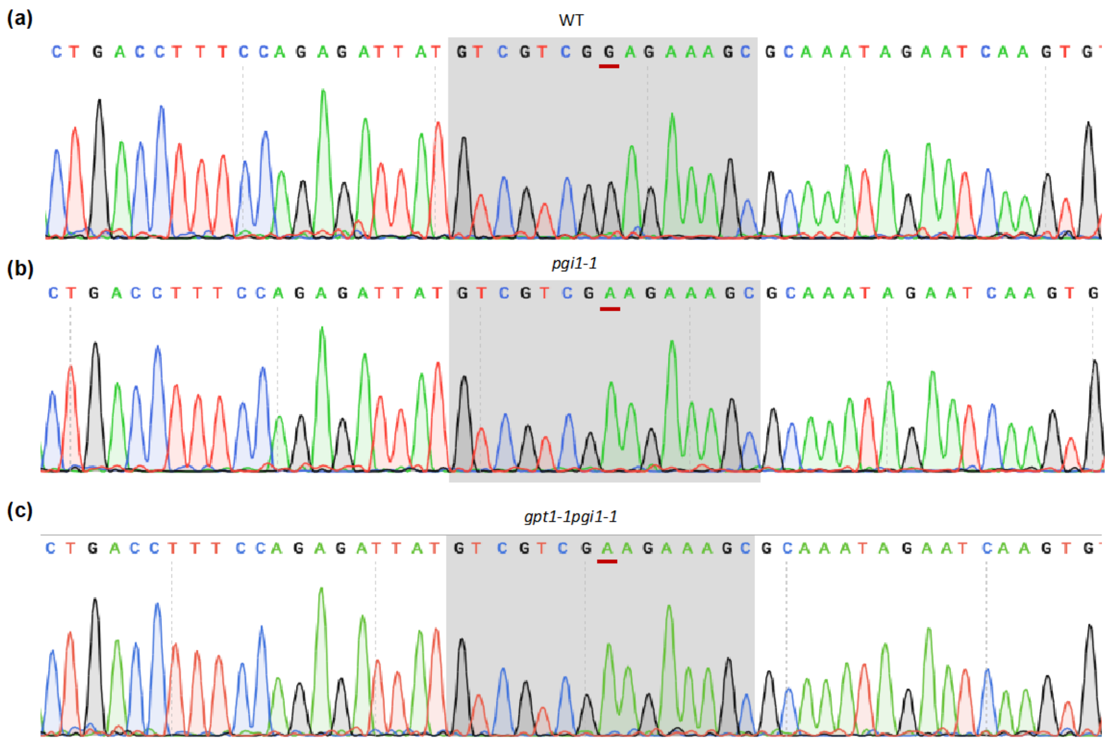

Supplemental Figure S1. Genotyping of *pgi* and *gpt1pgi* mutants.

Representative sequencing chromatograms of PCR amplicons around the base at position 834 (for primers see Supplemental Table S7). *Pgi* mutants have a point mutation from C to T at position 834, which is labelled in red in the complementary strands visualized in the chromatograms. (a) wild type, (b) *pgi* and (c) *gpt1pgi*.

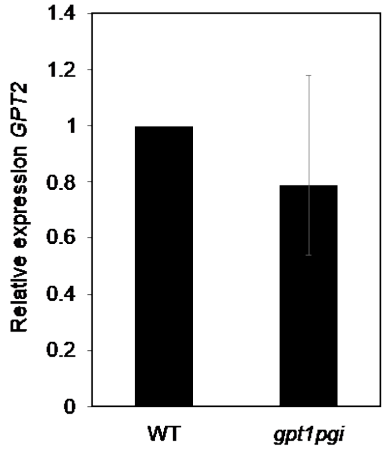

Supplemental Figure S2. Gene expression of *GPT2* in *gpt1pgi* mutants.

GPT2 gene expression in guard cell-enriched epidermal peels of wild-type and *gpt1pgi* plants at the end of night. Data from two independent experiments are shown; means ± fold change range; n = 6. *ACT2* was used as a housekeeping gene for normalization. For details about fold change and error calculations refer to Materials and Methods section. Primer sequences and efficiencies are given in Supplemental Table S7. No statistical differences were detected among genotypes for $P < 0.05$ determined by one-way ANOVA with post hoc Tukey's test.

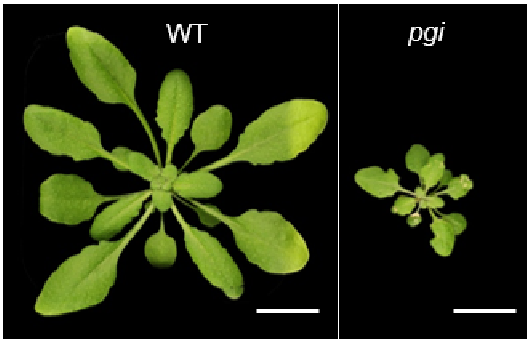

Supplemental Figure S3. Growth retardation of *pgi* mutants.

Images of 4-week-old wild-type and *pgi* plants grown under a 12 h light/12 h dark photoperiod. Scale bar = 1 cm. Frame WT = 4.42 cm^2. Frame *pgi* = 4.42 cm x 2.48 cm.

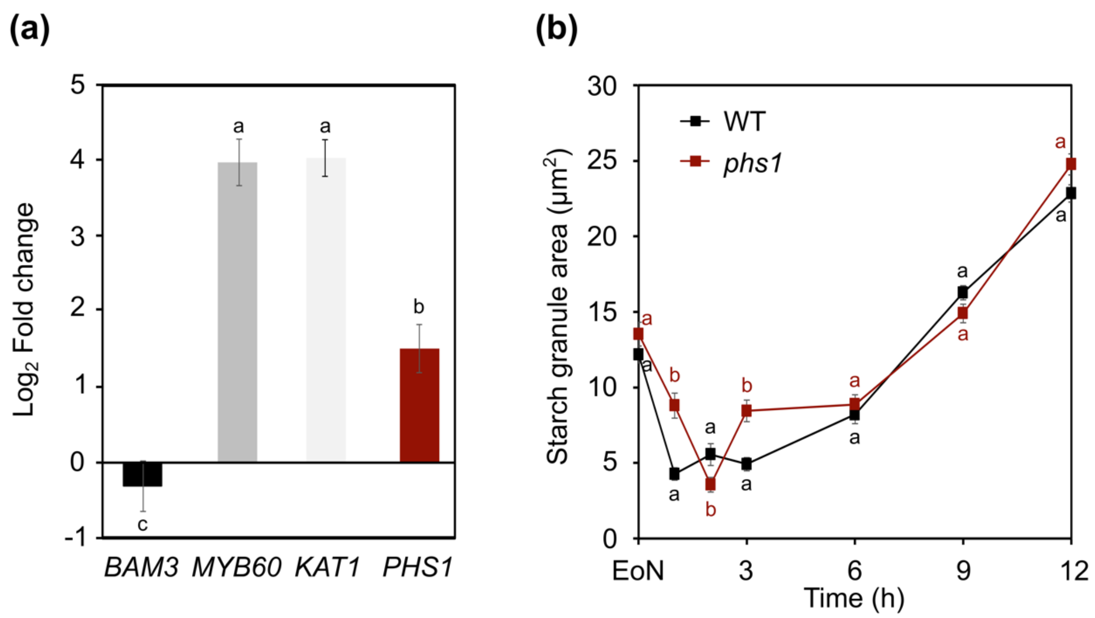

Supplemental Figure S4. Guard cell gene expression of *PHS1* and starch contents in *phs1* mutants.

(a) *PHS1* gene expression in wild-type guard cell-enriched epidermal peels relative to wild-type intact rosette leaves at EoN. *KAT1* and *MYB60* were used as markers for guard cell-specific expression, while *BAM3* was used as a leaf-specific marker. Data for the marker genes are the same as in Figure 2a. Data from two experiments are shown; means ± fold change range; n = 6. *ACT2* was used as a housekeeping gene for normalization. For details about fold change and error calculations refer to Materials and Methods section. Primer sequences and efficiencies are given in Supplemental Table S7. Letters indicate significant statistical difference between genes for $P < 0.05$ determined by one-way ANOVA with post hoc Tukey's test. (b) Starch dynamics in guard cells of intact leaves of WT and *phs1* plants over the 12 h light period. Plants were illuminated with 150 µmol m^{-2} s^{-1} of white light. Data from four independent experiments are shown; means ± SEM; n = 160 individual guard cells per genotype and time point. EoN = end of night. Wild type data are the same as in Figure 5b and 5d. Letters indicate significant statistical differences between genotypes for the given time point for $P < 0.05$ determined by one-way ANOVA with post hoc Tukey's test. WT = wild type.

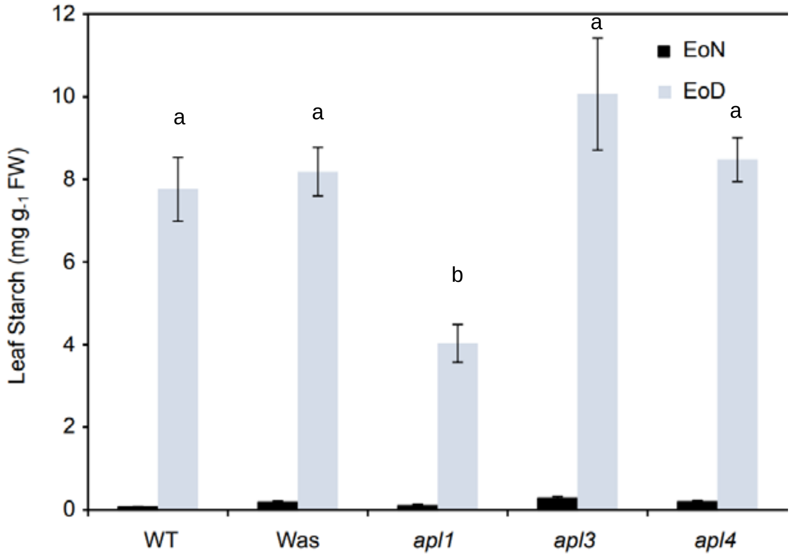

Supplemental Figure S5. Leaf starch contents in *apl* mutants.

Leaf starch amounts in wild-type (Col-0), Was, *apl1, apl3* and *apl4* mutant plants at the end of the night (EoN) and end of the day (EoD). Data from one experiment are shown; means ± SEM; n = 8. Letters indicate significant statistical difference between genotypes for the given time point for $P < 0.05$ determined by one-way ANOVA with post hoc Tukey's test.

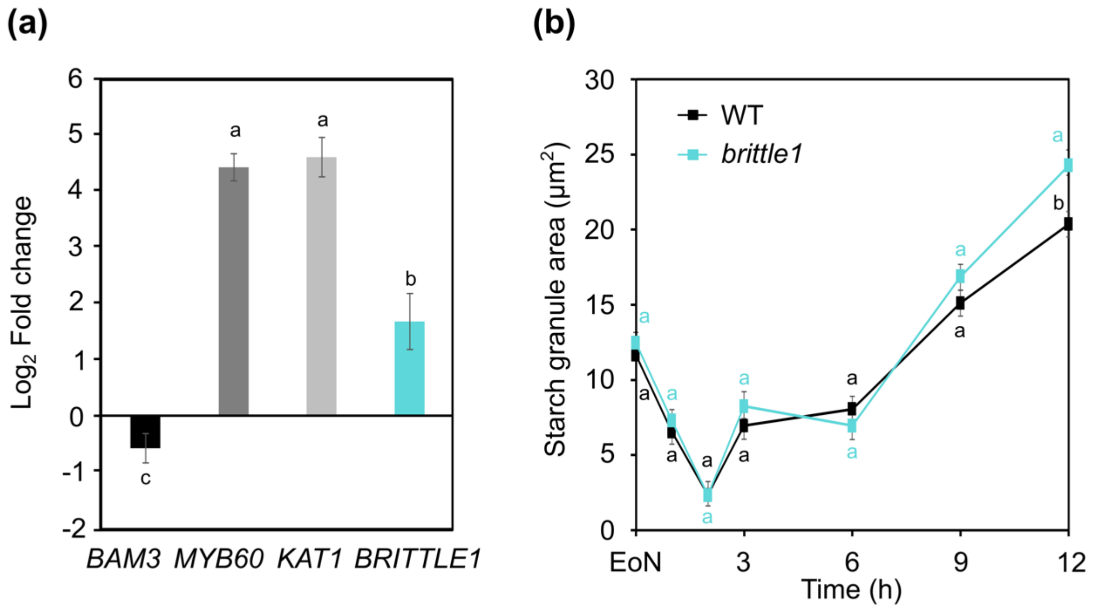

Supplemental Figure S6. Guard cell gene expression of *BRITTLE1* and starch contents in *brittle1* mutants.

(a) *BRITTLE1* gene expression in wild-type guard cell-enriched epidermal peels relative to wild-type intact rosette leaves at EoN. *KAT1* and *MYB60* were used as markers for guard cell-specific expression, while *BAM3* was used as a leaf-specific marker. Data from two experiments are shown; means ± fold change range; n = 6. *ACT2* was used as a housekeeping gene for normalization. For details about fold change and error calculations refer to Materials and Methods section. Primer sequences and efficiencies are given in Supplemental Table S7. Letters indicate significant statistical difference between genes for $P < 0.05$ determined by one-way ANOVA with post hoc Tukey's test. (b) Starch dynamics in guard cells of intact leaves of WT and *brittle1* plants over the 12 h light period. Plants were illuminated with 150 µmol m^{-2} s^{-1} of white light. Data from four independent experiments are shown; means ± SEM; n = 160 individual guard cells per genotype and time point. EoN = end of night. Wild type data are the same as in Figure 5b and 5d. Letters indicate significant statistical differences between genotypes for the given time point for $P < 0.05$ determined by one-way ANOVA with post hoc Tukey's test. WT = wild type.

Supplemental Table S1. Starch synthesis rates of wild-type, *pgi and gpt* mutant guard cells. Starch synthesis rates are derived from the slope in between time points. WT = wild type.

**Starch synthesis rates**										
**Genotype**	**Time points**									
	EoN - 1h light	1h light- 2h light	2h light- 3h light	3h light- 6h light	6h light- 9h light	9h light- 12h light	12h light- 15h light	15h light- 18h light	18h light – 21h light	21h light – 24h light
WT	-0.61	-0.21	1.27	0.65	0.20	0.002	0.09	0.003	0.03	-0.51
pgi	-0.56	-0.30	0.13	0.46	1.18	0.45	0.18	-0.08	-0.09	-0.56
gpt1	-0.82	0.44	1.00	0.44	0.59	0.08	0.34	0.01	-0.06	-0.62
gpt2	-0.62	0.63	-0.35	1.38	0.38	0.05	1.26	0.005	0.006	-0.58
gpt1pgi	-0.56	-0.47	-0.11	2.00	0.79	-0.06	0.38	-0.04	0.02	-0.42

Supplemental Table S2. Starch synthesis rates of wild-type and *gpt1gpt2* guard cells. Starch synthesis rates are derived from the slope in between time points. WT = wild type.

**Starch synthesis rates**						
**Genotype**	**Time points**					
	EoN-1h light	1h light- 2h light	2h light- 3h light	3h light- 6h light	6h light- 9h light	9h light- 12h light
WT	-0.58	-0.24	0.84	1.16	0.61	0.19
gpt1gpt2	-0.64	-0.05	-0.08	1.53	0.08	0.25

Supplemental Table S3. Starch synthesis rates of wild-type and *PGI* silencing lines guard cells. Starch synthesis rates are derived from the slope in between time points. WT = wild type.

**Starch synthesis rates**						
**Genotype**	**Time points**					
	EoN-1h light	1h light- 2h light	2h light- 3h light	3h light- 6h light	6h light- 9h light	9h light- 12h light
WT	-0.52	-0.28	0.67	1.01	0.18	0.27
amiR-*PGI* #1	-0.28	0.16	0.06	0.83	0.23	0.42
amiR-*PGI* #2	-0.39	-0.22	0.62	0.93	-0.10	0.85

Supplemental Table S4. Starch synthesis rates of wild-type and *PGM* silencing lines guard cells. Starch synthesis rates are derived from the slope in between time points. WT = wild type.

Starch synthesis rates						
**Genotype**	**Time points**					
	EoN-1h light	1h light-2h light	2h light-3h light	3h light-6h light	6h light-9h light	9h light-12h light
WT	-0.59	-0.53	2.69	0.68	0.14	0.27
amiR-*PGM* #1	-0.57	-0.15	0.21	0.13	0.32	1.44
amiR-*PGM* #2	-0.44	-0.30	0.18	-0.32	0.43	1.68

Supplemental Table S5. Starch synthesis rates of wild-type and *g1pt* mutant guard cells. Starch synthesis rates are derived from the slope in between time points. WT = wild type.

Starch synthesis rates						
**Genotype**	**Time points**					
	EoN-1h light	1h light-2h light	2h light-3h light	3h light-6h light	6h light-9h light	9h light-12h light
WT	-0.24	-0.41	0.53	0.61	0.28	0.09
g1pt1	0.02	-0.08	-0.17	0.41	-0.10	0.05
g1pt2	-0.32	-0.09	0.32	1.12	-0.12	0.41

Supplemental Table S6. Starch synthesis rates of wild-type, *phs1* and *apl* mutant guard cells. Starch synthesis rates are derived from the slope in between time points. WT = wild type.

Starch synthesis rates						
**Genotype**	**Time points**					
	EoN-1h light	1h light-2h light	2h light-3h light	3h light-6h light	6h light-9h light	9h light-12h light
WT	-0.64	0.30	-0.11	0.67	0.98	0.40
apl1	-0.75	0.61	0.23	0.83	0.47	0.49
apl4	-0.45	0.07	0.29	0.14	0.95	0.15
Was	-0.89	1.84	-0.11	1.71	0.15	0.98
apl3	-0.44	0.29	0.02	0.08	0.81	0.21
apl3apl4	-0.70	-0.74	2.96	0.79	1.08	0.37
phs1	-0.34	-0.59	1.37	0.05	0.67	0.66

Supplemental Table S7. Oligonucleotides used in this study.

gene	AGI code	forward primer	reverse primer	PCR efficiency	source
		**qPCR PRIMERS**			
ACT2	AT3G18780	CGTACAACCGGTATTGTGCT	GTAATCAGTAAGGTCACGTCCA	2.18	Flütsch *et al.*
BAM3	AT4G17090	TGATTCTGTGCCTGTCCT	GAATTTCCGCAATAACTCCTC	2.07	Horrer *et al.*
KAT1	AT5G46240	AGCATGGGATGGGAAGAGTGGAG	AGAGCAGTGTCGGAAGTCGGAT	1.88	Horrer *et al.*
MYB60	AT1G08810	CATGAAGATGGTGATCATGAGG	TTCCATTTGACCCCCAGTAG	1.98	Horrer *et al.*
GPT1	AT5G54800	GGCTGTTGGGATCGTTGAGA	GCCACAGCAACCGGAAAAAG	1.90	This study
GPT2	AT1G61800	CGAAGCAGTGAGGATGGTTT	TCACCGGAATGTTCTCTCCT	1.85	Bates *et al.*
PGI	AT4G24620	GGGATTAATGTTAGGGAGATG	TGTTACCGTCAAGATCAAACT	2.06	Bahaji *et al.*
PGM	AT5G51820	ACAGACCCTGTTGATGGA	TGTATATTCGCACTGTAGCTC	1.93	This study
PHS1	AT3G29320	CGACATTCAGATCAAGCGT	CTCTCACTAGCACTCATTTCC	1.95	This study
APL1	AT5G19220	TTTATACAGGATCATCGGCAGAG	TTGTGTCTACTGCCATTGCT	1.95	This study
APL3	AT4G39210	TGGCATAAACTTTGGAGATGG	CTTAGCATCCTCAAACACCC	1.91	This study
APL4	AT2G21590	GCTGAGAAATGTCGGATGG	ACATCAATGTATCCTGAAGCTC	1.98	This study
BRITTLE1	AT4G32400	CTGGAGTTAGCCAGACACTCTTG	TGCAGTGCTCGATAGTGCACCTG	1.99	This study
G1PT1	AT1G34020	CGTGGATGTTCAATGTCGTC	ACGGAAGATGAGAAGGCTGA	1.90	This study
G1PT2	AT4G09810	GGTAATGCCTCGTCCAAGCCAGGCGGC	AGCCCCAAGTTTCTGTTGTCGGGTCGT	2.04	This study
		**GENOTYPING PRIMERS**		**line**	
GPT1	AT5G54800	TTGACATACTCACCGTTGCAG	TCTCTCCCAGTATATACGCGC	SALK_021762	SIGnAL
GPT2	AT1G61800	CTTCATGGGAGAGACTTTCCC	TGATCTCACCGGAATGTTCTC	GABIKAT_454H06	SIGnAL
APL1	AT5G19220	GGAGCAGGAACTCGGCTC	TCTACCTCGGAAAGCCAA	EMS	SIGnAL
APL3	AT4G39210	GGAGGTTTCACATTCTCTCCC	TCCTATAGCATCCACCCACAG	FLAG_458A07	SIGnAL
APL4	AT2G21590	CGAATTAGGACCTCAAGGGTC	GTGATCTCTTATGGCTGCAGG	SALK_108632	SIGnAL
PHS1	AT3G29320	TCCACCGTTTCTTACCATCTG	GAACCGAAAGC CAAAGTAACC	GABI_257A06	SIGnAL
BRITTLE1	AT4G32400	CTCCATCTTCTCGTTGCTTTG	GAGGAAGGACCCACAGAACTC	SALK_026943	SIGnAL
LBb1.3		ATTTTGCCGATTTCGGAAC			SIGnAL
FLAG		CTACAAATTGCCTTTTCTTATCGAC			SIGnAL
GABIKAT Lb		GGGCTACACTGAATTGGTAGCTC			SIGnAL
		**CLONING PRIMERS**			
PGM amiRNA miR-S	AT5G51820	FWD - GATAAAGTAACGACCATCAGCTATCTCTCTTTTGTATTCC			WMD3
PGM amiRNA miR-A	AT5G51820	REV - GATAGCTGATGGTCGTTACTTTATCAAAGAGAATCAATGA			WMD3
*PGM amiRNA miR*S*	AT5G51820	FWD - GATAACTGATGGTCGATACTTTTTCACAGGTCGTGATATG			WMD3
*PGM amiRNA miR*A*	AT5G51820	REV - GAAAAAGTATCGACCATCAGTTATCTACATATATATTCCT			WMD3
PGI amiRNA miR-S	AT4G24620	FWD – GATGTATCTGAGTAAAACGCCCCTCTCTCTTTTGTATTCC			WMD3
PGI amiRNA miR-A	AT4G24620	REV - GAGGGGCGTTTTACTCAGATACATCAAAGAGAATCAATGA			WMD3
*PGI amiRNA miR*S*	AT4G24620	FWD - GAGGAGCGTTTTACTGAGATACTTCACAGGTCGTGATATG			WMD3
*PGI amiRNA miR*A*	AT4G24620	REV - GAAGTATCTCAGTAAAACGCTCCTCTACATATATATTCCT			WMD3
amiRNA A outside fwd		CTGCAAGGCGATTAAGTTGGGTAAC			WMD3
amiRNA B outside rev		GCGGATAACAATTTCACACAGGAAACAG			WMD3
		**SEQUENCING PRIMERS**		**line**	
amiRNA A outside fwd		CTGCAAGGCGATTAAGTTGGGTAAC			WMD3
amiRNA B outside rev		GCGGATAACAATTTCACACAGGAAACAG			WMD3
Sequencing of pgi1-1	AT4G24620	ACAAACAAAATTGAGTGGGAGT		TSY254	This study

Supplemental Table S8. Plasmids generated in this study.

Plasmid	Description	Restriction Enzymes	Fwd Primer	Rev Primer
MIR319a	pJET vector containing the artificial microRNA sequence	EcoRI, BamHI to release the artificial microRNA from pJET.	Please see Table S7 (all listed cloning primers)	Please see Table S7 (all listed cloning primers)
BJ36	Vector containing the *KST1* promoter for subcloning	EcoRI, BamHI to linearize the plasmid	M13 primers for sequencing	M13 primers for sequencing
BJ36-MIR319a	Vector carrying the artificial microRNA after the *KST1* promoter	NotI to release the insert	M13 primers for sequencing	M13 primers for sequencing
pART27	Vector for plant expression	NotI to linearize the plasmid	M13 primers for sequencing	M13 primers for sequencing
pSF10	Plant expression vector carrying the KST1 promoter upstream the artificial microRNA sequence against *PGM*			
pSF21	Plant expression vector carrying the KST1 promoter upstream the artificial microRNA sequence against *PGI*			

Supplemental References

Bahaji A, Sánchez-López ÁM, De Diego N, Muñoz FJ, Baroja-Fernández E, Li J, Ricarte-Bermejo A, Baslam M, Aranjuelo I, Almagro G, *et al.* 2015. Plastidic Phosphoglucose Isomerase Is an Important Determinant of Starch Accumulation in Mesophyll Cells, Growth, Photosynthetic Capacity, and Biosynthesis of Plastidic Cytokinins in Arabidopsis (DF Gomez-Casati, Ed.). *Plos One* **10**: e0119641.

Bates GW, Rosenthal DM, Sun J, Chattopadhyay M, Peffer E, Yang J, Ort DR, Jones AM. 2012. A Comparative Study of the Arabidopsis thaliana Guard-Cell Transcriptome and Its Modulation by Sucrose. *PLoS ONE* **7**.

Flütsch S, Nigro A, Conci F, Fajkus J, Thalmann M, Trtílek M, Panzarová K, Santelia D. 2020. Glucose uptake to guard cells via STP transporters provides carbon sources for stomatal opening and plant growth . *EMBO reports* **21**: 1–13.

Horrer D, Flütsch S, Pazmino D, Matthews JSA, Thalmann M, Nigro A, Leonhardt N, Lawson T, Santelia D. 2016. Blue Light Induces a Distinct Starch Degradation Pathway in Guard Cells for Stomatal Opening. *Current Biology*: 362–370.
